# Self-powered flexible Janus-like metal–organic framework membrane for sustainable moisture-enabled electrokinetic energy harvesting

**DOI:** 10.1039/d5ta06289f

**Published:** 2025-09-16

**Authors:** Amalia Rizki Fauziah, Flora Schöfbeck, Michael R. Reithofer, Jia Min Chin

**Affiliations:** a Institute of Functional Materials and Catalysis, Faculty of Chemistry, University of Vienna Währinger Straße 42 1090 Vienna Austria jiamin.chin@univie.ac.at; b Vienna Doctoral School in Chemistry (DoSChem), University of Vienna 1090 Vienna Austria; c Institute of Inorganic Chemistry, Faculty of Chemistry, University of Vienna Währinger Straße 42 1090 Vienna Austria

## Abstract

Amid growing demand for clean, affordable, and sustainable energy, leveraging naturally available resources such as atmospheric moisture has become increasingly attractive. In this work, we introduce MOFs@FP-CB, a flexible Janus-like asymmetric membrane developed through a straightforward dip-coating process and engineered for efficient electrokinetic energy harvesting from controlled humidity. A carbon-black-modified filter paper substrate is coated with two functional layers of the membrane, comprising a hydrophilic, hygroscopic, negatively charged SO_4_-MOF-808 layer on one side and a hydrophobic, positively charged ZIF-8 layer on the other side, arranged laterally. By creating a steady lateral moisture gradient, this asymmetric arrangement facilitates directed and selective ion transport *via* nanoconfined MOF channels. The device produces a voltage of 0.20 V and a current of 20.6 μA at controlled conditions (25 °C and 65% relative humidity (RH)). Electrical output can be easily scaled owing to its modular design, reaching up to 129.7 μA and 1.49 V through basic parallel and series connections, respectively, and we further showed that the system is capable of powering a red LED when 20 membranes are connected in series. The membrane provides exceptional mechanical flexibility and operational durability while maintaining its performance under a wide range of environmental conditions, regardless of temperature and RH. These characteristics position MOFs@FP-CB as a viable and affordable platform for next-generation wearable, self-powered moisture energy harvesting systems.

## Abstract 2

A self-powered, flexible, durable, and adaptable Janus MOFs-based membrane generates electricity from ambient humidity for next-generation sustainable wearable energy harvesters.

## Graphical abstract

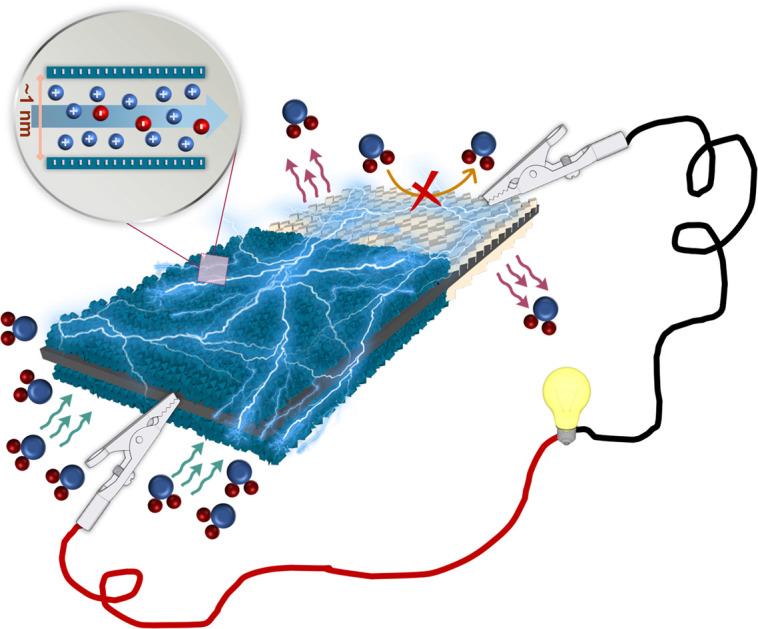

## 1. Introduction

With the increasing worldwide demand for clean energy and the urgent need for mitigating environmental deterioration, the development of sustainable energy technologies that harness abundant natural resources has become increasingly critical. Amongst the range of technologies, water-based energy harvesting is especially compelling, as water is not only sustainable and highly abundant but also exists in various forms throughout the environment. Strategies such as salinity gradient,^1–3^ electrokinetic systems,^4,5^ thermoelectric generators,^6–8^ wave^9,10^ and tidal energy,^11^ hydroelectric power,^12–15^ and moisture-driven electricity^16–19^ have attracted widespread interest. These approaches utilize the ubiquitous presence of water to generate electricity through mechanisms such as ion transport, fluid dynamics, and interfacial interactions, offering environmentally friendly solutions for sustainable energy conversion. Moisture-enabled energy generation (MEG) harnesses moisture from the air as a sustainable energy source by converting spontaneous moisture absorption and diffusion into electrical power, typically through asymmetric moisture gradients and surface charge interactions.^20,21^ This approach is particularly attractive and ideal for low-resource conditions, due to its zero liquid requirement, controlled operation, and ability to harvest energy from atmospheric moisture. However, primarily due to weak driving forces and poor ionic mobility in the moisture phase, it suffers from slow ion transport, low power density, and limited charge separation efficiency.^22–26^ On the other hand, electrokinetic energy generation (EKEG) that uses ion transport through porous material or charged nanochannels under pressure or concentration gradients shows more stable output performance, strong charge separation, and high ionic flux, particularly in aqueous environments.^27–31^ However, the use of conventional EKEG is limited under passive or dry conditions, as it typically requires bulk water, external pressure, or microfluidic flow.^32,33^ This hybrid system leverages continuous moisture uptake and directional transport, from the absorption side to the evaporative side, to create a self-sustaining water flow inside the membrane, thereby driving ion transport through confined, charged channels. This combination overcomes the transport constraints of traditional MEG and eliminates the requirement for external pressure in EKEG systems. Consequently, the system offers a scalable, effective, and sustainable solution for energy harvesting from moisture.

As reticular materials with highly ordered sub-nanochannels and pores, metal–organic frameworks (MOFs) offer precise control over the kinetics of water uptake and release, high surface area, tunable hydrophilicity or hydrophobicity, and adjustable porosity^34–37^ and have been widely used for energy harvesting.^38–40^ Lee *et al.* recently demonstrated a MEG system featuring a hydrophilic MOF that harnesses and releases moisture through temperature-driven day–night cycling.^41^ As a result, energy can be generated through condensation on a conductive Ni-HHTP MOF. Our system, in contrast, applies a Janus-like MOF with asymmetrical properties to produce a hydrophilicity gradient, enabling continuous self-powered operation independent of temperature gradient, without the need for day–night cycling. Together with electrokinetic effects, this design increases ionic mobility, promotes directional water flow, and guarantees a steady energy output, demonstrating its potential as an efficient MEG device platform. To validate the concept, we developed a flexible Janus-like asymmetric membrane (MOFs@FP-CB) that integrates moisture absorption and electrokinetic ion transport for continuous energy harvesting. Given MOFs' attractive properties, two MOFs with complementary characteristics are chosen for use as both the absorbing and evaporating sections in such an MEG system. The SO_4_-MOF-808 acts as an effective hygroscopic agent,^42,43^ drawing moisture from the air, while ZIF-8's hydrophobic,^44^ porous structure facilitates efficient water evaporation. Together, their arrangement enhances electrokinetic energy harvesting without the need for bulk water by maintaining a moisture flux that drives the movement of water and ions through the highly ordered, nanochannels of the MOFs.

**Fig. 1:**
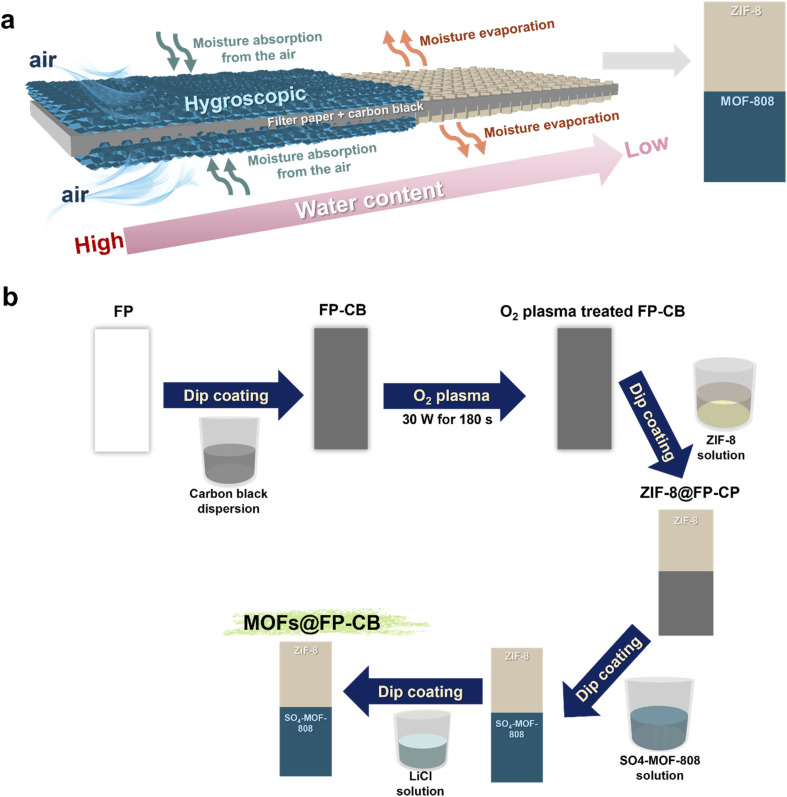
MOF-based moisture-enabled energy generation membrane design. (a) The MOF-based membrane exhibits Janus-like properties, featuring a hygroscopic, hydrophilic surface on one side and a hydrophobic surface on the other. (b) Schematic of the fabrication process for the MOFs@FP-CB membrane *via* a dip-coating method.

A carbon black-coated filter paper (FP-CB) substrate is asymmetrically coated with a hygroscopic, hydrophilic SO_4_-MOF-808 layer on one end and a hydrophobic ZIF-8 layer on the other end (Fig. 1a). Unlike vertical-gradient MEG systems, which often suffer from transient output limited to humidity changes,^22^ strong environmental dependence with narrow relative humidity (RH) tolerance,^45^ bulky and less scalable architectures,^46^ rapidly dissipating gradients due to the vertical structure, leading to short operational durations,^47^ and reliance on specialized materials and complex fabrication methods that restrict versatility,^48,49^ this unique lateral charge and wettability asymmetry establishes a persistent in-plane water and ionic gradient, which not only supports continuous self-powered operation but also enhances directional ion selectivity and streaming potential *via* nanoconfined electrokinetic channels. It produces 20.6 μA of current and 0.20 V of voltage under controlled conditions (25 °C, 65% RH), resulting in 1.08 μW of power. The device's potential for wearable applications is demonstrated by its stable performance even when bent. Electrical output can be scaled through basic circuit configurations. A parallel connection of 10 membranes increases current to 129.7 μA, while a series of 12 membranes boosts voltage to 1.49 V. Finally, as a proof-of-concept, the MOFs@FP-CB device was demonstrated to power a red-light emitting diode (LED) as an example of a miniature electronic device. The synergistic function of the SO_4_-MOF-808 layer (moisture absorption and ion transport), FP-CB substrate (water transport), and ZIF-8 layer (moisture evaporation) enables sustained, self-driven electricity generation for up to 168 h by establishing selective and directional ion transport in confined MOFs' channels. Fabricated *via* a simple dip-coating process using low-cost materials, the MOFs@FP-CB membrane offers an affordable, electrode-free platform for practical, flexible, autonomous, and scalable moisture energy harvesting.

## 2. Experimental section

### 2.1 Materials

All chemicals and materials used were commercially available and their detailed information were summarized as below: filter paper (qualitative, Whatman 5) was purchased from Cytiva, carbon black, (Lot: Q04I024; particle size: 42 nm) acetylene, 50% compressed (99.9%) and zirconium(iv) chloride (ZrCl_4_, 99.5%) were supplied by Thermo Scientific. Cetyltrimethylammonium bromide (C_19_H_42_BrN; CTAB, 98%) and 1,3,5-benzenetricarboxylic acid (C_9_H_6_O_6_, 98%) were provided by Alfa Aesar. Zinc nitrate hexahydrate (Zn(NO_3_)_2_·6H_2_O, 98%), 2-methylimidazole (C_4_H_6_N_2_, 99%), acetic acid (CH_3_COOH, ≥99.8%), isopropanol (C_3_H_8_O, ≥99.8%), and formic acid (HCOOH, 100%) were received from Sigma-Aldrich. Sulfuric acid (H_2_SO_4_, 95%) was obtained from Fisher Chemical. Absolute ethanol (C_2_H_5_OH, 100%) was produced by Chem-Lab. All solvents used were of analytical grade, and all chemicals were utilized as received without further purification. Deionized water (resistivity: 18.2 MΩ cm) used throughout the experiments was supplied by a Millipore Direct-Q system.

### 2.2 Synthesis of SO_4_-MOF-808

MOF-808 nanoparticles were prepared by adopting the synthesis method described by Dai *et al.*^50^ ZrCl_4_ (200 g, 0.84 mol) was added to a mixture of acetic acid (300 mL) and isopropanol (500 mL) under stirring at 500 rpm, then heated at 120 °C for 60 min. The resulting Zr_6_ oxocluster product was collected by centrifugation at 10 000 rpm (10 956 rcf), washed twice with acetone, and dried under vacuum at room temperature. Subsequently, 0.6 g of Zr_6_ oxocluster was dispersed in formic acid (3 mL) under stirring at 600 rpm, followed by the addition of H_2_O (5 mL) until the mixture became completely colorless. Then, 1,3,5-benzenetricarboxylic acid (150 mg, 0.7 mmol) was added, and the reaction mixture was stirred overnight. The resulting solid was collected by centrifugation at 15 000 rpm (24 652 rcf) for 5 min, washed sequentially with 40 mL of deionized water and ethanol, and dried under vacuum for 3 h to give a final product of white MOF-808 powder. Subsequently, 0.5 g of the synthesized MOF-808 was treated with 50 mL of 0.1 mol per L H_2_SO_4_ under magnetic stirring for 24 h.^51^ The white solid was collected by centrifugation, washed three times with deionized water and absolute ethanol, and finally dried in a vacuum oven at 120 °C for 12 h, yielding SO_4_-MOF-808.

### 2.3 Fabrication of ZIF-8 powder

ZIF-8 crystals were synthesized *via* a modified rapid mixing method in aqueous solution at room temperature.^52^ Typically, 0.744 g (2.5 mmol) of Zn(NO_3_)_2_·6H_2_O dissolved in 10 mL of deionized water was rapidly added to a 2-methylimidazole solution (12.3 g, 150 mmol) in 90 mL of deionized water under stirring. The mixture was stirred for 5 min and then left undisturbed at room temperature for 24 h. The resulting ZIF-8 crystals were collected by centrifugation at 10 500 rpm, washed three times with deionized water, and dried overnight at 50 °C.

### 2.4 Preparation of MOFs solution

40 mg each of SO_4_-MOF-808 and ZIF-8 powders were suspended in 20 mL of absolute ethanol, forming solutions of SO_4_-MOF-808 and ZIF-8, respectively, which were then used to dip-coat the FP-CB substrate.

### 2.5 Synthesis of carbon black dispersion

The carbon black (CB) dispersion was prepared using a literature-known procedure.^53^ Initially, 200 mg of cetyltrimethylammonium bromide (CTAB, C_19_H_42_BrN) was dissolved in 20 mL of a 1 : 1 (v/v) ethanol–water mixture and sonicated for 5 min to ensure complete dissolution. Subsequently, 400 mg of CB was introduced into the CTAB solution, followed by ultrasonication for 30 min and magnetic stirring for an additional 30 min to achieve a homogeneous dispersion.

### 2.6 Fabrication of MOFs@FP-CB MEG membrane

Rectangular Whatman filter paper (FP) was dip-coated with CB dispersion for several seconds, dried at 80 °C for 15 min, and treated with O_2_ plasma (30 W, 180 s) to produce a conductive membrane, referred to as FP-CB. An asymmetric Janus-like membrane (MOFs@FP-CB) was fabricated by sequentially dip-coating SO_4_-MOF-808 and ZIF-8 onto opposite ends of the FP-CB substrate for several seconds, with coating lengths of 2.5 cm and 1.5 cm, respectively, unless otherwise specified. Each coating step was followed by thermal drying at 80 °C for 3 min. Finally, the SO_4_-MOF-808 side of MOFs@FP-CB was selectively dip-coated in 1 M LiCl solution to introduce additional alkali ions into the system before electrical measurements.

### 2.7 Material characterizations

The crystalline phases of the MOFs were characterized by X-ray diffraction (XRD) using a high-throughput Empyrean Panalytical (Malvern) diffractometer operating in reflection mode, equipped with a Cu-Kα radiation source (*λ* = 1.5406 Å) and a PIXcel3D detector with a current of 40 mA and voltage of 45 kV. Data were collected at a scan rate of 2° min^−1^ with a step size of 0.01°. Zeta potential measurements were conducted in absolute ethanol (0.2 mg mL^−1^) at neutral pH using a Malvern Zetasizer Nano ZS equipped with a disposable folded capillary cell (DTS1070). The Brunauer–Emmett–Teller (BET) specific surface area and pore size distribution of the MOFs were determined from nitrogen adsorption–desorption isotherms measured at 77 K using a Quantachrome iQ2 instrument (Anton Paar) gas adsorption analyzer. Prior to analysis, the samples were degassed under vacuum at 150 °C for 3 h. The morphological structure and elemental composition of the membranes were examined using field-emission scanning electron microscopy (FESEM; Zeiss Supra 55 VP) coupled with X-ray spectroscopy (EDX), operated in high vacuum mode at an acceleration voltage of 1–15 kV. Samples were mounted on conductive carbon tape and sputter-coated with a ∼10 nm gold layer to minimize charging effects.

### 2.8 Electrical measurement

The MEG performance of the designed membrane, including open-circuit voltage (*V*_OC_, hereafter referred to as voltage) and short-circuit current (*I*_SC_, hereafter referred to as current), was recorded using a Metrohm Autolab NOVA 2.1.2. Unless otherwise specified, measurements were conducted at 25 °C and 65% RH to simulate practical conditions, with temperature and humidity precisely controlled by a Binder humidity chamber.

### 2.9 Scale-up of MOFs@FP-CB

The current and voltage outputs of the MOFs@FP-CB membrane can be effectively enhanced by connecting multiple units in series or parallel configurations, respectively, using crocodile clips to establish electrical contact between them.

### 2.10 Stability test of MOFs@FP-CB

The MOFs@FP-CB membrane was kept in a humidity-controlled chamber at 25 °C and 65% RH throughout the data acquisition period for subsequent stability testing.

## 3. Results and discussion

### 3.1 MOF-based electrokinetically driven moisture energy generator fabrication

The design of the proposed flexible Janus-like MOF-based MEG device is schematically illustrated in Fig. 1a. The membrane consists of a fibrous FP-CB, which acts as both a conductive substrate and water transport layer. The substrate is asymmetrically coated with two MOFs of contrasting properties. On one side, a layer of SO_4_-MOF-808, which is hygroscopic, hydrophilic, and negatively charged, was used, serving as the moisture-absorbing layer. On the other side, a ZIF-8 layer, which is hydrophobic and positively charged, was used, acting as the evaporative layer. This design produces a laterally asymmetric Janus-like MEG membrane, termed MOFs@FP-CB, which establishes a horizontal water and ion content gradient. During operation, selective ion transport occurs through the nanometer-scale channels of the MOF layers, driven mainly by the internal ion concentration gradient from the SO_4_-MOF-808 side to the ZIF-8 side. Concurrently, undissociated water molecules flow laterally through the porous, conductive FP-CB substrate *via* capillary forces and electrokinetic effects. These coordinated movements create an internal ion flux and an external electron flow across the asymmetric membrane, producing streaming currents and voltages that can be harvested as electrical energy, which were measured using a simple setup with the membrane clamped by crocodile clips at both ends (Fig. S1).

SO_4_-MOF-808 was prepared at room temperature through a multi-step process involving the synthesis of (i) a zirconium oxocluster (ZrO_6_), (ii) MOF-808, and (iii) subsequent sulfation to obtain SO_4_-MOF-808, as illustrated in Fig. S2 (see Experimental section). Meanwhile, ZIF-8 was synthesized in aqueous solution at room temperature (see Experimental section and Fig. S3). Both MOF powders were dispersed in absolute ethanol at a concentration of 2 mg mL^−1^ as dip-coating solutions (Fig. S4) to fabricate the MOF-based MEG membrane, referred to as MOFs@FP-CB. In contrast to previous studies that relied on complex synthesis methods,^46,54–56^ our MOFs@FP-CB (Fig. 1a) was fabricated *via* a simple and versatile dip-coating technique using low-cost, widely available materials of CB and FP as substrate (Fig. 1b). A round Whatman FP was first cut into a 1 cm × 4 cm rectangular strip (Fig. S5a) and subsequently dip-coated with a conductive CB dispersion to introduce electrical conductivity.^57^ The CB dispersion was prepared by dispersing CB powder in a 1 : 1 ethanol–water mixture as illustrated in Fig. S6a (see details in the Experimental section), followed by ultrasonication and stirring to obtain a stable dispersion lasting at least 12 h (Fig. S6b). The coated membrane was then oven dried at 80 °C to obtain a FP-CB membrane with uniform surface coverage (Fig. S5b), followed by O_2_ plasma treatment at 30 W for 180 s to introduce hydrophilic oxygen-containing groups,^58–61^ such as carboxyl (–COOH) and hydroxyl (–OH), onto the FP-CB surface, as illustrated in Fig. S7. The electrically conductive FP-CB membrane, rich in hydrophilic groups, was subsequently coated with MOFs *via* dip-coating, with SO_4_-MOF-808 serving as the moisture-absorbing layer on one lateral side and ZIF-8 as the water-evaporating layer on the other. The resulting membrane is compact (1 cm × 4 cm), lightweight (83.8 mg), and flexible, as shown digitally in Fig. S8.

### 3.2 The material characterizations of MOFs for moisture-driven energy harvesting membrane

**Fig. 2:**
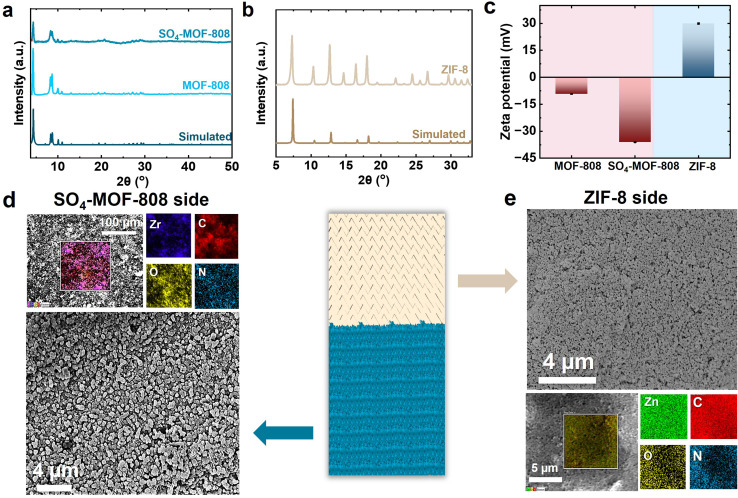
Material characterization of the metal–organic frameworks used in the moisture-enabled energy generation membrane. The X-ray diffraction patterns of (a) SO_4_-MOF-808 and (b) ZIF-8 closely match their respective simulated pattern, confirming successful synthesis. (c) ZIF-8 and SO_4_-MOF-808 hold opposite charges. The field-emission scanning electron microscopy images show (d) SO_4_-MOF-808 coated on one side of the carbon black-coated filter paper substrate and (e) ZIF-8 on the opposite lateral side of the membrane.

We characterized the MOFs by XRD, zeta potential, and N_2_ adsorption–desorption isotherms to confirm their successful synthesis. The XRD patterns shown in Fig. 2a and b, along with their theoretical structural model (Fig. S9), reveal the highly ordered crystalline structures of the MOFs, which provide uniform and well-defined channels for ion transport.^62–64^ The XRD patterns of MOF-808 before (light blue) and after sulfation (dark blue) show that the SO_4_-MOF-808 pattern closely resembles both the simulated and pristine MOF-808 patterns (Fig. 2a). Characteristic peaks at 4.3°, 8.3° and 8.7°, corresponding to the (111), (311) and (222) planes, along with additional reflections at 10.0° and 11.0°, confirm the high crystallinity.^65–67^ The identical peak positions further imply the retained structural integrity of the framework following SO_4_^2−^ incorporation. For ZIF-8 (Fig. 2b), the prominent peak at 7.4° corresponds to the (011) plane, indicative of its truncated rhombic dodecahedron morphology. Additional reflections at 10.2°, 12.5°, and 18° attributed to the (002), (112), and (222) miller indices,^68,69^ respectively, consistent with the simulated pattern. The surface charge polarity of the MOFs was determined by zeta potential measurements in absolute ethanol at neutral pH. MOF-808 displayed a negative value of −9.25 mV (Fig. 2a and S10), which was caused by deprotonated hydroxyl and terminal carboxylate groups on its zirconium clusters.^70^ The successful incorporation of SO_4_^2−^ and the presence of sulfate groups are confirmed by the significantly higher negative zeta potential (−36.10 mV) of SO_4_-MOF-808 upon sulfation as displayed in Fig. 2c and S10. The confinement of highly negatively charged SO_4_^2−^ enhances hydrophilicity^71^ and the cation selectivity of MOF-808. In contrast, because of its imidazole groups,^72^ ZIF-8 holds a positive zeta potential (+30 mV) as presented in Fig. 2c and S10. This lateral distribution of opposite surface charges across the membrane facilitates directional ion transport and efficient charge separation by enhancing the internal electric field and moisture-driven energy conversion.^73,74^ Fig. S11a and S12 display the N_2_ adsorption–desorption isotherms of SO_4_-MOF-808 and ZIF-8, respectively, revealing high Langmuir surface areas calculated *via* the BET method, indicative of abundant microporous channels that facilitate selective and rapid ion transport,^75,76^ thereby enhancing moisture-driven energy conversion. Fig. S11a and b show that the BET surface area and pore volume of MOF-808 decreased from 902.82 m^2^ g^−1^ and 0.60 cm^3^ g^−1^ for pristine MOF-808 to 512.43 m^2^ g^−1^ and 0.31 cm^3^ g^−1^ for SO_4_-MOF-808, respectively. This decrease is most likely the result of sulfate groups partially occupying the pores. Additionally, Fig. S11b demonstrates that MOF-808 and SO_4_-MOF-808 both possess distinct nanometer-scale channels with a highly ordered window-cavity architecture, which is typical of MOFs and effective for selective ion transport.

The membrane morphologies were characterized by FESEM to evaluate their structural features. The substrates transition from bare FP (Fig. S13a) to FP-CB, shown both before (Fig. S13b and S14a) and after (Fig. S13c and S14b) O_2_ plasma treatment. The FP surface was uniformly coated with CB, and no significant morphological changes were observed post-treatment, indicating that the plasma process (30 W, 180 s) did not compromise the coating. Instead, it strengthened the C and O signals (Fig. S14). The successful asymmetric coating of FP-CB is confirmed by FESEM images in Fig. 2d and e, where a uniform SO_4_-MOF-808 layer is present on one side and ZIF-8 is present on the other side. It can also be seen that the SO_4_-MOF-808 maintains its crystalline morphology following SO_4_^2−^ incorporation (Fig. 2d). Zr and Zn elements were found to be uniformly distributed in the SO_4_-MOF-808 (Fig. 2d) and ZIF-8 (Fig. 2e) regions, respectively, as confirmed by energy-dispersive EDX elemental mapping analyses. Additionally, the presence of sulfur (S) detected on the SO_4_-MOF-808 side (Fig. S15), otherwise absent in pristine MOF-808 (Fig. S16), further confirms the successful sulfation of MOF-808.

Integrating MOF nanochannels with opposite zeta potential and hydrophilicity properties on the lateral ends of the FP-CB substrate generates internal electrostatic and moisture gradients that synergistically drive directional water and ion transport under humid conditions, establishing an internal electrochemical gradient that enhances ion mobility while suppressing random diffusion. Strong interactions between charge carriers and the MOF functional groups allow for size- and charge-based selective ion sieving within these confined pathways. Consequently, the structural asymmetry of MOFs@FP-CB plays a critical role in boosting the overall current and voltage output, energy conversion efficiency, and operational stability of the MEG system.

### 3.3 The intrinsic factors governing the performance of MOFs@FP-CB

The output performance of the MOFs@FP-CB membrane was systematically evaluated under controlled temperature and humidity conditions. The fibrous FP-CB substrate acts as both a mechanical support for the MOF layers and a capillary-driven water transport medium, mimicking plant transpiration by generating spontaneous capillary pressure at the wet/dry interface under a water gradient (Fig. S17). The asymmetric surface modification with hygroscopic, hydrophilic (Fig. S18), negatively charged SO_4_-MOF-808 (Fig. 2c) and hydrophobic (Fig. S18), positively charged ZIF-8 (Fig. 2c) establishes an internal ionic gradient that drives the directional migration of hydrated counterions (*e.g.*, H^+^, alkali metal cations) through MOF-confined nanochannels, enabling selective ion diffusion and conduction based on surface charge (Fig. S19 and S20a). Simultaneously, undissociated water molecules, preserved due to the low degree of autoionization at controlled conditions (*K*_w_ ≈ 10^−14^ at 25 °C),^77,78^ are transported laterally through the porous FP-CB substrate as illustrated in Fig. S20a *via* capillary action, flowing from the SO_4_-MOF-808 side (moisture uptake layer) to the ZIF-8 side (evaporation layer), where they are released. This coupled and continuous water-ion transport generates a net electrokinetic potential, which drives the migration of cations present in the Stern and diffuse layers in the hydrophilic SO_4_-MOF-808 side towards the ZIF-8 side, producing stable streaming voltage and ionic current under controlled conditions (Fig. S20b).

**Fig. 3:**
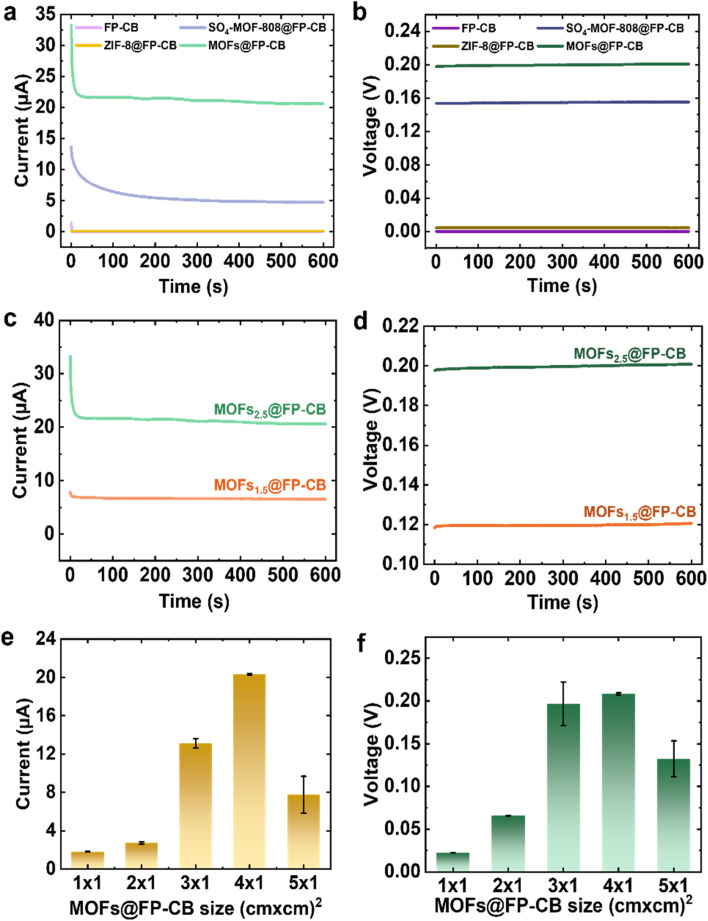
Internal factors affecting the moisture energy generation performance of the MOFs@FP-CB membrane. (a and b) Composition, (c and d) ratio, and (e and f) size effects on the generated current and voltage, respectively.

Five membranes with different compositions (as listed in Table S1) were fabricated and tested to elucidate the role of each membrane component. The pristine FP substrate was unable to absorb moisture, as visualized in Fig. S21a, which shows that it produced no detectable current of 0 μA (Fig. S22). A minor improvement in performance was noted upon the addition of the O_2_ plasma-treated conductive CB layer, which produced a small current of 0.02 μA (Fig. 3a and S23a) and a voltage of 0.115 mV (Fig. 3b and S23b). This improvement is attributed to the introduction of hydrophilic functional groups, such as –COOH and –OH, and to the conductive CB surface, which slightly improves moisture absorption and facilitates electron conduction pathways. However, the overall moisture absorption remained insufficient as schematically depicted in Fig. S21b, thus constraining ion mobility and resulting in low electrical output. Further modification of the FP-CB membrane by coating one side with hydrophobic ZIF-8 resulted in only a marginal improvement in electrical performance by 0.06 μA and 4.5 mV (Fig. 3a, b and S23), suggesting enhanced moisture evaporation but still limited moisture uptake due to ZIF-8's low water affinity and lack of strong hygroscopic and hydrophilic interactions (Fig. S21c). These findings demonstrate how crucial the hygroscopic and hydrophilic SO_4_-MOF-808 layer is for improving environmental moisture uptake (Fig. S24) and creating a horizontal water content gradient across the membrane (Fig. S17), which promotes efficient directional ion transport by forming distinct wet and dry regions. According to Fig. 3a and b, for SO_4_-MOF-808@FP-CB and MOFs@FP-CB, the voltage is increased by 33.3-fold and 44.4-fold and the current is enhanced by ∼78.5-fold and ∼343.3-fold, respectively, in comparison to ZIF-8@FP-CB.

Comparing the Janus-like MOFs@FP-CB membrane to the SO_4_-MOF-808@FP-CB membrane, the former achieved a significantly higher current of 20.60 μA and voltage of 0.20 V, while the latter achieved 4.71 μA and 0.15 V. The MEG performance of MOFs@FP-CB is therefore improved 4.37 times in current and 1.33 times in voltage when compared to the SO_4_-MOF-808@FP-CB membrane. In the absence of the ZIF-8 evaporative layer, the SO_4_-MOF-808@FP-CB membrane relies only on the FP-CB substrate's capillary action for moisture desorption, resulting in slow and inefficient evaporation (Fig. S17a). This occurs because the treated FP-CB not only facilitates moisture evaporation but also absorbs a small amount of moisture from the air (Fig. S24a) due to its hydrophilic groups. As moisture absorption dominates, near-complete wetting occurs (Fig. S17a), disrupting the ionic gradient essential for MEG. On the other hand, the Janus-type MOFs@FP-CB membrane with an integrated SO_4_-MOF-808 absorbing layer and ZIF-8 evaporative layer maintains a steady wet/dry interface (Fig. S17b), thereby driving hydrated counterions in an advective, directional flux toward the drier ZIF-8 side. The hierarchical pore structure of the SO_4_-MOF-808 layer (∼18 Å cavities connected by ∼6 Å windows) allows for effective moisture uptake and bears highly ordered nanochannels rich in counter-cations. Its strongly negative surface potential (−36.1 mV), introduced *via* sulfation, improves ion dissociation and electrostatically attracts mobile cations into the channels. By contrast, the ZIF-8 layer suppresses bulk water condensation while preserving an internal humidity and electrostatic gradient (Fig. S24b), due to its smaller pore apertures (∼3.4 Å), hydrophobic cage-like structure, and positive surface charge (+30 mV). ZIF-8 sustains the internal gradient that drives and directs ion migration, thereby enhancing electrical output. This built-in asymmetry in surface charge and wettability establishes a horizontal gradient in water content and electrostatic potential, sustaining a persistent, cation-dominated streaming current and thereby maximizing the membrane's electrokinetic energy output under a humidity gradient.

To examine the necessity of O_2_ plasma treatment of FP-CB prior to MOF coating, we compared untreated and O_2_ plasma-treated MOFs@FP-CB membranes. Fig. S25 presents how the treated membrane produced noticeably higher current and voltage outputs than the untreated one, which only produced 8.37 μA and 0.13 V. The performance enhancement is attributed to the introduction of abundant –COOH and –OH functional groups on the FP-CB membrane surface *via* O_2_ plasma treatment.^61^ These groups release protons (H^+^) and produce a negative surface charge when they partially dissociate into –COO^−^ and –O^−^ species in humid environments. This treatment simultaneously enhances membrane hydrophilicity and cation selectivity, facilitating the directional transport of hydrated cations along the internal humidity gradient. As a result, ion migration is accelerated, and MEG performance is improved. To better understand the individual roles of each MOF layer, we investigated whether the moisture absorbing SO_4_-MOF-808 or the evaporative ZIF-8 plays a more dominant role in electricity generation. To this end, we fabricated two membranes with varied layer ratios. One with a longer SO_4_-MOF-808-coated side (MOFs_2.5_@FP-CB, Fig. S26a) and the other with a longer ZIF-8-coated side (MOFs_1.5_@FP-CB, Fig. S26b), with dimensions detailed in Table S2. Fig. 3c and d show that MOFs_2.5_@FP-CB delivers superior performance compared to MOFs_1.5_@FP-CB, which generated only 6.53 μA and 0.12 V, implying that SO_4_-MOF-808 as the absorbing layer governs the MEG process more than the evaporative ZIF-8 layer. Extending the absorbing layer lowers internal resistance, promotes sustained charge transport, improves ion conductivity, moisture uptake and retention, and raises output voltage and current. An excessively long evaporating layer, on the other hand, may lead to too much water evaporation, disrupting ion migration and reducing output performance.

To further optimize the MEG device, the performance of MOFs@FP-CB membranes was evaluated as a function of membrane length (from 1 to 5 cm), with a fixed width of 1 cm (Fig. S27 and Table S3). The corresponding current outputs for MOFs@FP-CB_1_ to MOFs@FP-CB_5_ were 1.82, 2.72, 13.1, 20.6, and 7.76 μA, respectively (Fig. 3e), while the generated voltages were 0.02, 0.06, 0.197, 0.20, and 0.132 V (Fig. 3f). Among them, MOFs@FP-CB_4_ exhibited the highest output, delivering a harvested power of 1.08 μW (calculated using eqn (S1)) as presented in Fig. S28a and b and an extracted power density of 0.27 μW cm^−2^ (calculated using eqn (S2)) as shown in Fig. S28c and d. See the SI for calculation details. These results indicate an optimal membrane length at 4 cm, as performance declines at both shorter and longer lengths.

Overly long membranes limit output by increasing ionic resistance, reducing ion flux, and weakening the moisture gradient, as both ion migration and water transport occur over longer lateral distances. Ion mobility is restricted, and current output is reduced by localized evaporation or saturation imbalances, which further impede continuous water flow, whereas membranes which are too short may not harvest sufficient moisture for ion generation. To understand the influence of MOF membrane thickness, primarily governed by the number of coating cycles, and vertical ion transport pathways on overall device performance, the functional thickness of the MOF membrane was systematically optimized. We varied the number of dip-coating cycles (Table S4), thereby modulating the MOF layer thickness on the FP-CB substrate. The current output tends to weaken with increasing thickness, as confirmed in Fig. S29a. As a thicker MOF layer lengthens the vertical transport path, diffusion limitation increases ionic resistance and reduces total current generation. In contrast, a noticeable enhancement in voltage was observed (Fig. S29b). This improvement is attributed to greater moisture absorption and ion accumulation within the thicker MOF layers, which enhances the ion concentration gradient and extends the ion separation pathway, resulting in a strengthened internal electric field.

These findings highlight the need for careful optimization of membrane length and thickness to achieve a favorable balance between enhanced current and voltage output and minimal transport resistance, ensuring effective electrokinetic performance. Overall, the rectangular membrane with dimensions of 4 cm × 1 cm, featuring a single coating of SO_4_-MOF-808 as the moisture absorbing layer and ZIF-8 as the evaporative layer on laterally opposite sides of an O_2_ plasma-treated FP-CB membrane, and designed with a longer absorbing layer to create a horizontal wet–dry interface, represents the optimal MEG device configuration. This design achieves superior MEG performance, generating 20.60 μA and 0.20 V, surpassing previous membranes in both current (Fig. S30a) and voltage output (Fig. S30b), as summarized in Table S5, and will be used for further electrical evaluation.

### 3.4 Highly adaptable MOFs@FP-CB membranes for external environmental stimuli

**Fig. 4:**
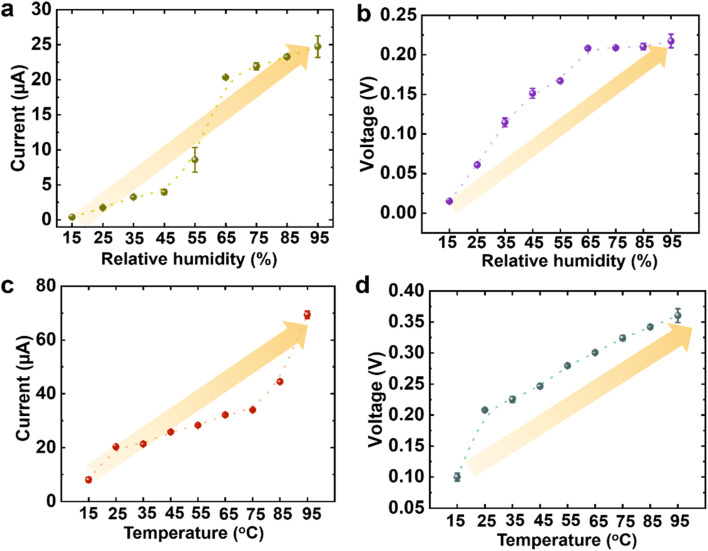
Environmental adaptability of the MOFs@FP-CB membrane. The as-developed membrane functions effectively across a wide range of (a and b) relative humidity and (c and d) temperature conditions.

It is essential to comprehend how environmental factors, particularly temperature and % RH, affect MOF-based MEG performance. Temperature regulates evaporation rate and ion mobility, whereas % RH controls membrane hydration and moisture gradient stability. Together, these variables control the horizontal wet–dry interface in the MOFs@FP-CB membrane, promoting effective charge conduction and water transport. To systematically assess these effects, RH was varied from 15% to 95% at a constant temperature of 25 °C to isolate its role in moisture absorption and ionic conductivity. Conversely, temperature was adjusted from 15 °C to 95 °C under a fixed RH of 65% to evaluate its impact on evaporation rate and ion transport efficiency. The setpoints of 25 °C and 65% RH were selected as they simulate common controlled conditions, providing insight into device behavior under realistic environmental scenarios. Fig. 4a, b and S31 reveal that at 25 °C, both the current and voltage output of MOFs@FP-CB membrane rise from 0.38 μA and 0.015 V to 24.7 μA and 0.22 V as the % RH increases from 15 to 95, with the internal resistance decreasing to 8.3 kΩ under 95% RH (Fig. S32a). Similar trends were also observed when we altered the temperature and fixed the % RH. The current and the voltage gradually enhanced from 8.08 to 69.37 μA (Fig. 4c and S33a) and 0.1 to 0.36 V (Fig. 4d and S33b) at 15 to 95 °C respectively, at constant % RH of 65 and the calculated internal resistance of the membrane decreases to as low as 5.28 kΩ as the temperature increases to 95 °C, as presented in Fig. S32b. Due to the complex interplay between moisture uptake, evaporation rate, and internal membrane resistance, the results show that improvements in current and voltage are not linearly proportional to changes in temperature or % RH, although increasing % RH leads to increased moisture absorption, thus increasing the concentration of mobile ions within the membrane, which improves ionic conductivity and promotes charge separation. Furthermore, the devices' performance improves as the temperature rises because of improved ion mobility and accelerated water diffusion. These findings further demonstrate the robust adaptability of the MOFs@FP-CB membrane, maintaining stable performance across a wide range of temperatures (cold to hot) and humidity levels (dry to wet). Additionally, the robustness of the MOFs@FP-CB membrane under a range of environmental exposures may also be attributed to its resistance against airborne pollutants. MOFs@FP-CB membrane systems may be disrupted by airborne pollutants like CO_2_, SO_2_, and volatile organic compounds (VOCs) through surface fouling, competitive binding, or adsorption. Nevertheless, both SO_4_-MOF-808 and ZIF-8 exhibit excellent chemical stability. SO_4_-MOF-808, featuring robust Zr_6_ nodes, retains its structural integrity under humid SO_2_ conditions.^79^ ZIF-8 likewise withstands acidic environments and captures volatile species without structural degradation.^80^ Additionally, under realistic environmental conditions, typical aqueous pollutants such as dissolved salts, organic solutes, and acidic or basic species have minimal impact on ion transport and device performance, due to the vapor-phase water harvesting mechanism and the intrinsic chemical robustness of SO_4_-MOF-808 and ZIF-8. Further, the hydrophobic ZIF-8 layer reduces moisture-assisted acidic gas uptake while the acid resistance of SO_4_-MOF-808 guarantees unaffected ion transport and water sorption.

### 3.5 Scalable and flexible self-powered MOFs@FP-CB membranes

**Fig. 5:**
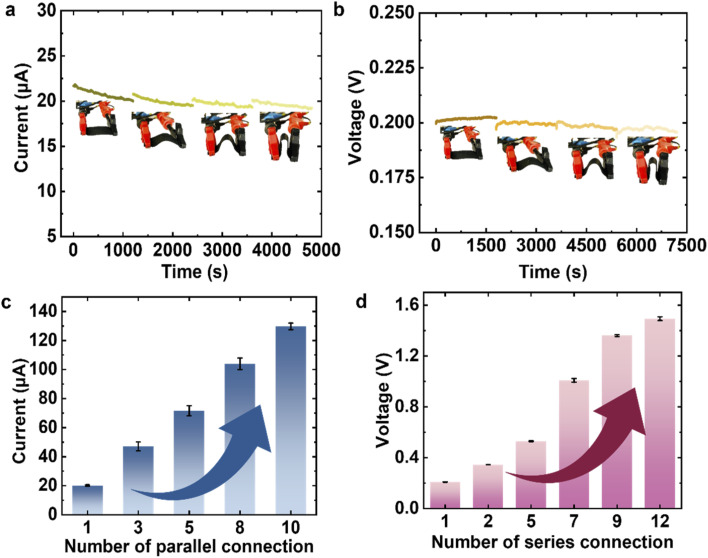
The practical applicability of the MOFs@FP-CB membrane. The membrane maintains (a) generated current and (b) extracted voltage under various bending angles, and its output can be scaled up through (c) parallel and (d) series connections.

We also demonstrated the flexibility, scalability, and sustainability of the MOFs@FP-CB membrane. MOFs@FP-CB retains stable current and voltage outputs even when bent at various angles, comparable to its performance in the unbent state, as shown in Fig. 5a and b. The flexible (Fig. S8b), fibrous structure (Fig. S13a) of the FP-CB substrate accommodates bending without compromising electrical performance or the attachment between MOFs and FP-CB substrate (Fig. S34). Notably, due to their nanoscale thickness, strong interfacial adhesion, and conformal growth along the fibrous network, the thin layers of SO_4_-MOF-808 and ZIF-8 grown on the substrate remain intact with negligible cracking, preserving continuous ion transport pathways even under mechanical stress. This exceptional flexibility demonstrates the membrane's suitability for integration into wearable electronics, providing a sustainable and viable option for portable energy harvesting applications.

The overall power output can be increased by integrating multiple MOFs@FP-CB membrane-based MEG devices, with current output scaled *via* parallel connections (Fig. S35a) and voltage enhanced through series connections (Fig. S36a). As the number of membranes connected in parallel increases, the current rises (Fig. 5c and S35b), while the voltage increases with the number of membranes connected in series (Fig. 5d and S36b). When 10 MOFs@FP-CB membranes are connected in parallel, the output current rises to 129.7 μA—6.3 times that of a single membrane (20.6 μA; Fig. 5c and S35b). Likewise, connecting 12 membranes in series boosts the voltage from 0.20 V (single membrane) to 1.49 V—a 7.45-fold increase (Fig. 5d and S36b). By wiring 20 devices in series (see schematic, Fig. S37; photographs, Fig. S38a and b), we powered a red LED at 25 °C and 65% RH (Fig. S38c), which remained illuminated under ambient, uncontrolled conditions as shown in Fig. S38d. These results highlight the practical potential of MOFs@FP-CB membranes for energy harvesting and demonstrate that MOFs@FP-CB membranes can convert ambient humidity into a reliable power source for future electronic applications.

The practical applicability of the MOFs@FP-CB membrane was further assessed by evaluating both its operational and material stability. Operational stability was examined through long-term testing under controlled conditions (25 °C, 65% RH), with continuous monitoring of current–time (*I*–*t*) and voltage–time (*V*–*t*) responses. The output current at 0 V stayed constant over 6 h cycles for 3 days in a row, as shown in Fig. S39, exhibiting only a slight decrease from 20.6 to 16 μA (∼1.3-fold) over 72 h, demonstrating sustained current generation capability. Similarly, Fig. S40 shows that over 168 h, the membrane's output voltage decreased only slightly from 0.20 to 0.18 V at a constant current of 0 μA, corresponding to a reduction of approximately 1.16-fold. These results underline the membrane's potential for reliable long-term moisture energy harvesting due to its durable performance and stable electricity generation. Electrical measurements collectively confirm that the observed electrokinetic signals originate from asymmetric, moisture-driven ion conduction through confined MOF channels rather than electronic pathways. The bare FP-CB substrate generated negligible current under 25 °C and 65% RH (Fig. S22) and exhibited high resistance in the kilo-ohm range, confirming its insulating nature. Furthermore, the output of symmetric devices coated completely with ZIF-8 or SO_4_-MOF-808 (ZIF-8//ZIF-8 and SO_4_-MOF-808//SO_4_-MOF-808) was noticeably lower (Fig. S41a and b) in contrast to the configuration of the Janus type (Fig. 3a and b), emphasizing the crucial function of structural asymmetry and interfacial charge contrast in permitting directional ion transport.

In addition, the MOFs@FP-CB membrane possesses excellent morphological stability post-testing, fulfilling a critical requirement for practical applications. FESEM images show that following a 24 h operation at 25 °C and 65% RH, both the SO_4_-MOF-808 (Fig. S42a) and ZIF-8 (Fig. S42b) materials maintained their structural integrity. Each image inset displays EDX elemental mappings emphasizing the water stability of both MOFs, showing Zr and Zn signals that are still clearly discernible on the ZIF-8 and SO_4_-MOF-808 regions, respectively. The 24 h post-operation XRD patterns shown in Fig. S43 closely match those of the pristine MOFs, further confirming that both MOF structures retain their crystallographic integrity under humid operating conditions. The strong Zr–O cluster structure of SO_4_-MOF-808 contributes to its durability by offering remarkable chemical and hydrothermal resistance in moist conditions.^81,82^ Similarly, the sodalite-type structure of ZIF-8, reinforced by strong Zn–N coordination bonds, maintains structural integrity and offers inherent resistance to moisture-induced degradation.^83–85^

## 4. Conclusions

A flexible Janus-like asymmetric membrane, MOFs@FP-CB, composed of hygroscopic, hydrophilic SO_4_-MOF-808 and hydrophobic ZIF-8 layers laterally coated on low-cost, carbon-black-coated filter paper *via* a simple dip-coating method, was developed in this study. The asymmetric integration of oppositely charged MOFs on the lateral sides of the conductive FP-CB substrate establishes a stable, horizontal moisture gradient, enabling directional and selective ion transport through the confined channels of the MOFs. With 25 °C and 65% RH, this design achieves a high output of 20.6 μA and 0.20 V. The membrane's modular design allows straightforward scaling, reaching currents up to 129.7 μA in parallel and voltages up to 1.49 V in series arrangement. MOFs@FP-CB membrane holds outstanding adaptability over a broad range of temperatures and humidity along with mechanical flexibility and long-term stability. These results highlight its promise as a low-cost, durable, and scalable solution for moisture-driven energy harvesting in self-powered and wearable electronics.

## Conflicts of interest

The authors declare no conflicts of interest.

## Supplementary Material

TA-014-D5TA06289F-s001

## Data Availability

The data supporting this article have been included as part of the SI. See DOI: https://doi.org/10.1039/d5ta06289f.
