# Supplementary material for: Self-powered flexible Janus-like metal–organic framework membrane for sustainable moisture-enabled electrokinetic energy harvesting
Source: J Mater Chem A Mater. 2025 Sep 16;14(3):1571–83. doi: 10.1039/d5ta06289f (PMC12453016; doi:10.1039/d5ta06289f)
Supplement: TA-014-D5TA06289F-s001 [file TA-014-D5TA06289F-s001.pdf]

Supplementary Information (SI) for Journal of Materials Chemistry A.
This journal is © The Royal Society of Chemistry 2025

Supporting Information for

Self-powered flexible Janus-like metal-organic framework membrane for sustainable moisture-enabled electrokinetic energy harvesting

Amalia Rizki Fauziah,[a] *Flora* Schöfbeck,[a,b] *Michael R. Reithofer,*[c] *Jia Min Chin*[a,1*]

[a] Institute of Functional Materials and Catalysis, Faculty of Chemistry, University of Vienna, 1090 Vienna, Austria

[b]Vienna Doctoral School in Chemistry (DoSChem), University of Vienna, 1090 Vienna, Austria

[c]Institute of Inorganic Chemistry, Faculty of Chemistry, University of Vienna, 1090 Vienna, Austria

[1*]Corresponding Authors
Email: jiamin.chin@univie.ac.at (Jia Min Chin)

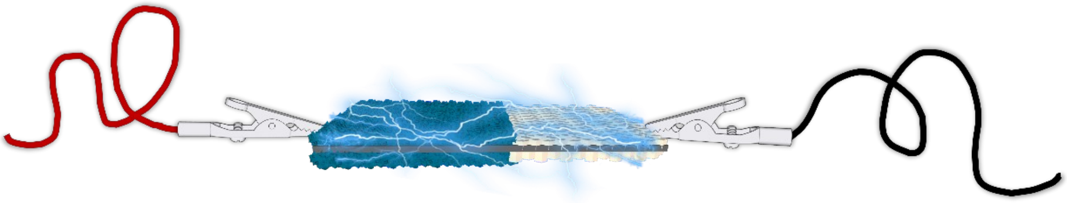

Fig. S1. Schematic illustration of the MOFs@FP-CB membrane-based device for moisture-enabled energy harvesting.

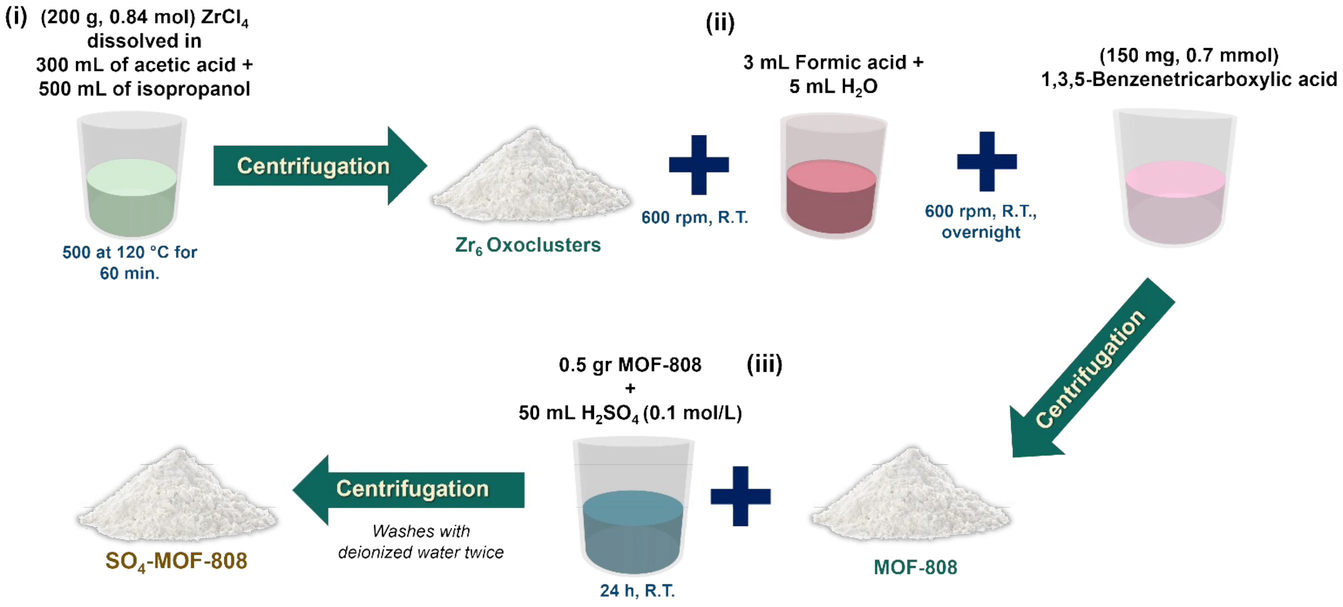

Fig. S2. Step-by-step illustration of the SO_4-MOF-808 powder synthesis process, consisting of (i) formation of Zr_6 oxocluster, (ii) synthesis of MOF-808, and (iii) final preparation of SO_4-MOF-808.

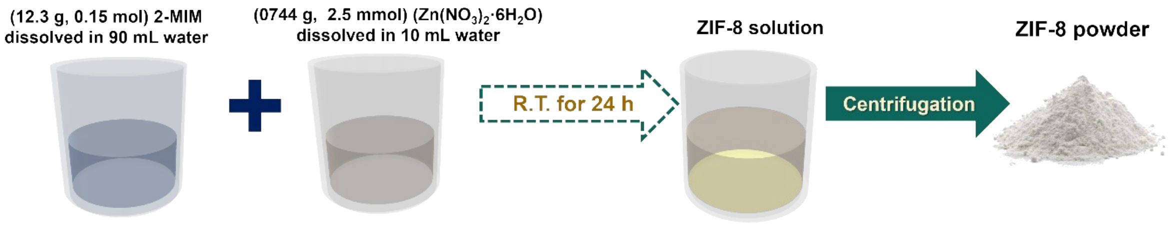

Fig. S3. Schematic illustration of the synthesis process for ZIF-8 powder.

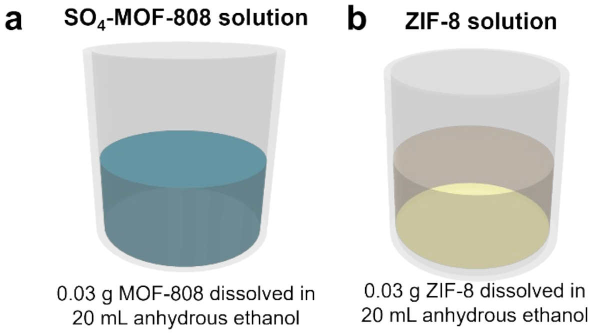

Fig. S4. Preparation of MOF solutions for coating the FP-CB substrate. (a) ZIF-8 and (b) SO_4-MOF-808.

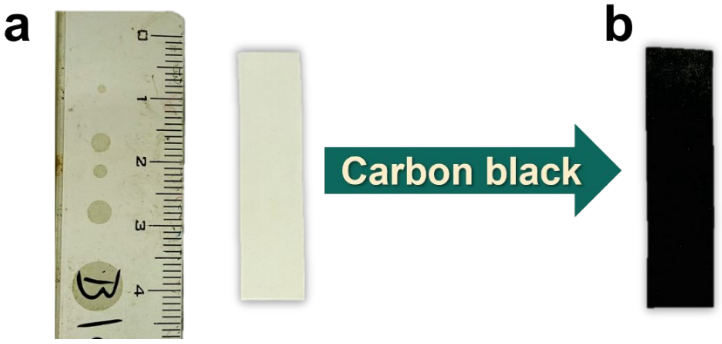

Fig. S5. Digital photographs of the filter paper substrate. (a) before and (b) after carbon black coating.

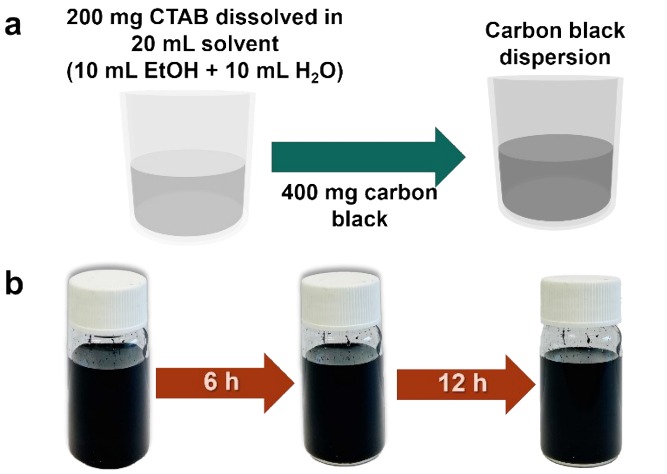

Fig. S6. Preparation of carbon black dispersion. (a) Schematic of the carbon black dispersion process. (b) The resulting dispersion forms a stable suspension that remains uniform for at least 12 h.

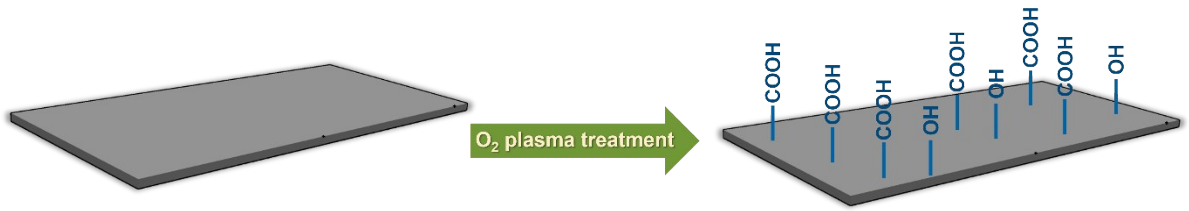

Fig. S7. O_2 plasma treatment introduces –COOH and –OH functional groups, enhancing the membrane's hydrophilicity.

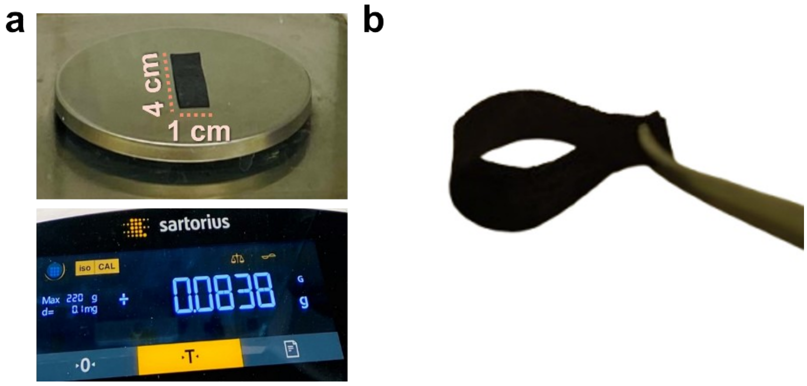

Fig. S8. Features of the as-developed MOFs@FP-CB membrane. (a) lightweight and (b) flexible.

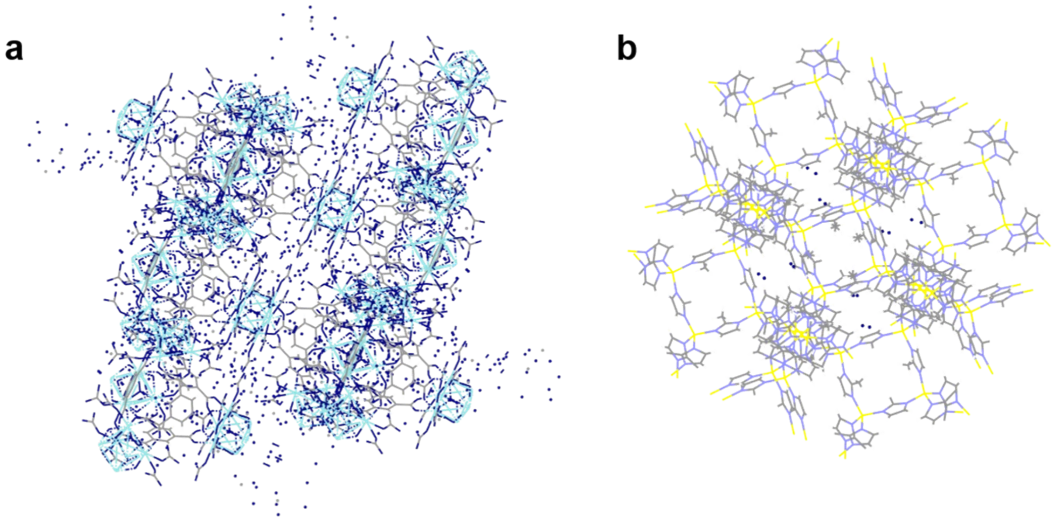

Fig. S9. The theoretical structural models of the (a) MOF-808 and (b) ZIF-8.

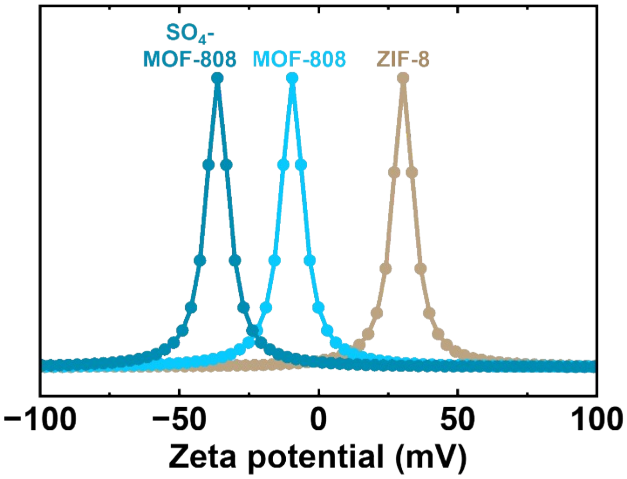

Fig. S10. Zeta potential analysis of the MOF components coated onto the MOFs@FP-CB membrane.

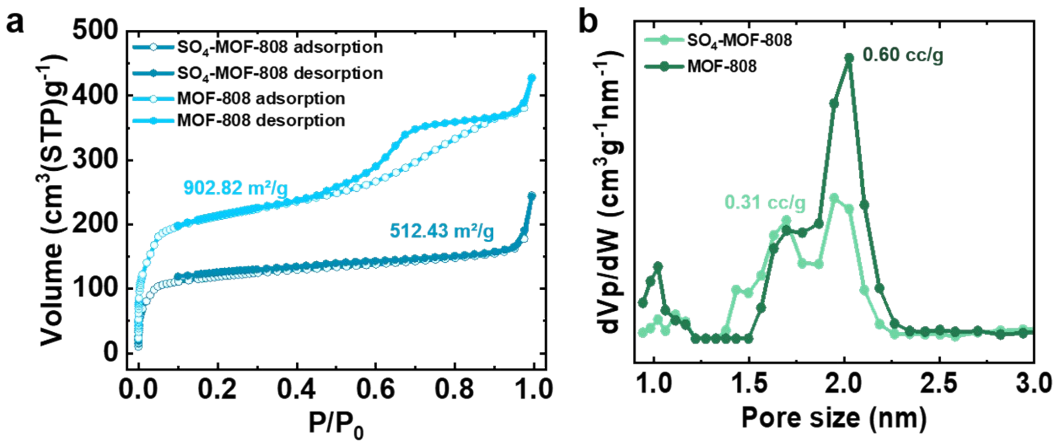

Fig. S11. N_2 adsorption-desorption isotherms of MOF-808 before (light color) and after (dark color) sulfation. (a) Surface area and (b) pore volume of MOF-808 decrease upon the introduction of the $–SO_4^{2-}$ functional group.

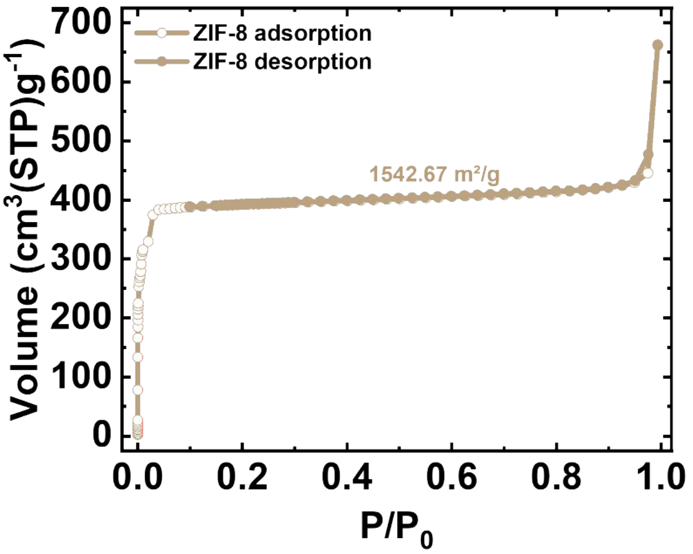

Fig. S12. BET surface area analysis of ZIF-8 based on N_2 adsorption-desorption measurements.

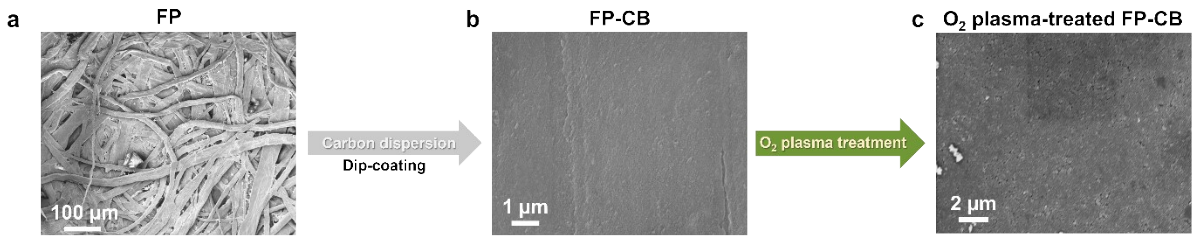

Fig. S13. Morphological transformation of the filter paper substrate. (a) pristine filter paper, (b) after carbon black coating, and (c) following subsequent O_2 plasma treatment.

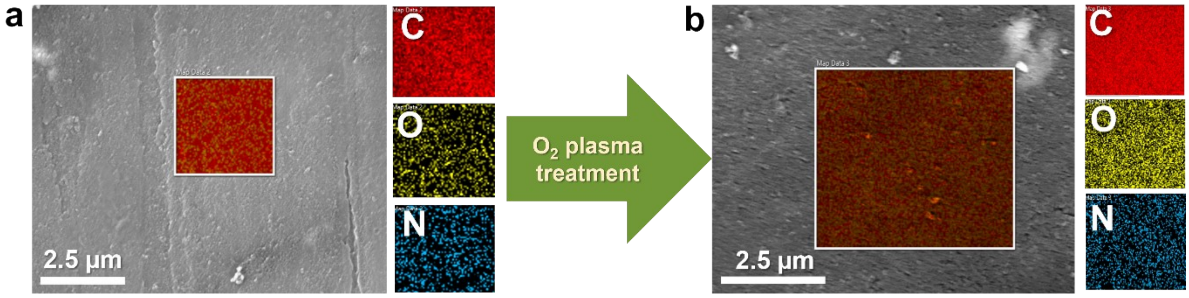

Fig. S14. The EDX mapping analysis of FP-CB (a) before and (b) after O_2 plasma treatment, showing an increase in carbon and oxygen content due to the plasma-induced surface modification.

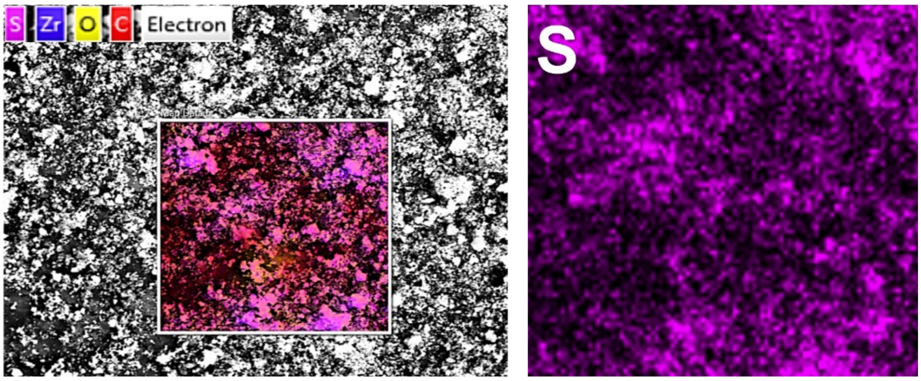

Fig. S15. Elemental mapping corresponding to Figure 2d confirms the presence of sulfur (S) element in the SO_4-MOF-808.

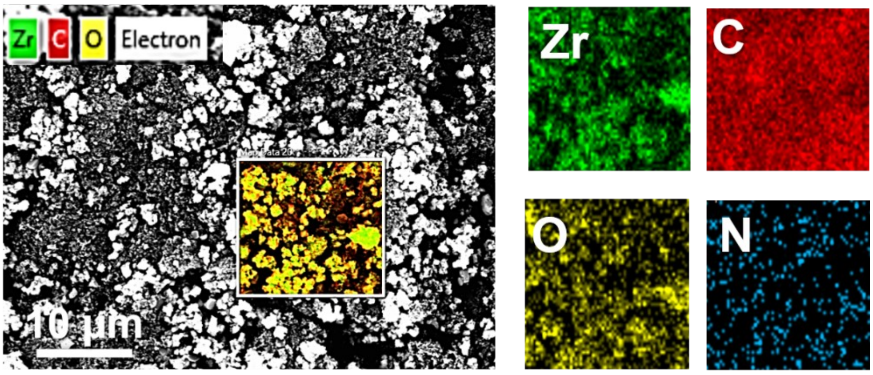

Fig. S16. The elemental EDX mapping analysis reveals no detectable sulfur (S) in pristine MOF-808.

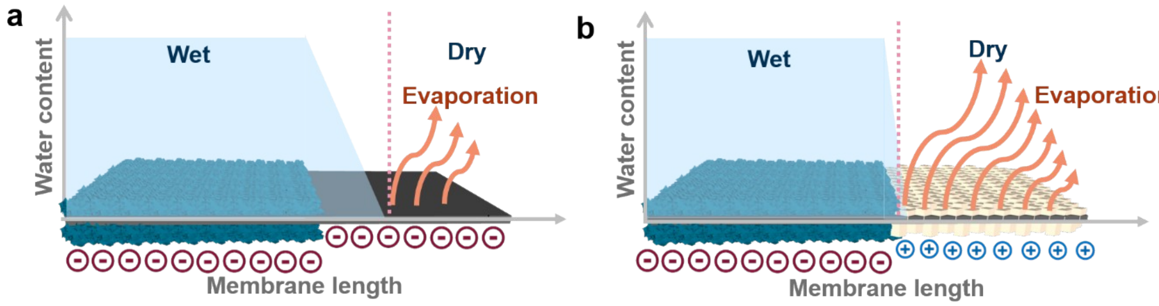

Fig. S17. Water content as a function of (a) SO_4-MOF-808@FP-CB and (b) MOFs@FP-CB membrane length.

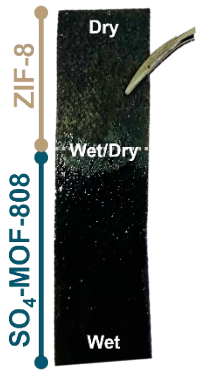

Fig. S18. Digital photograph of MOFs@FP-CB after exposure to 25 °C and 95% RH, illustrating the contrasting moisture responses. The hygroscopic and hydrophilic SO_4-MOF-808 at one end and hydrophobic ZIF-8 at the other.

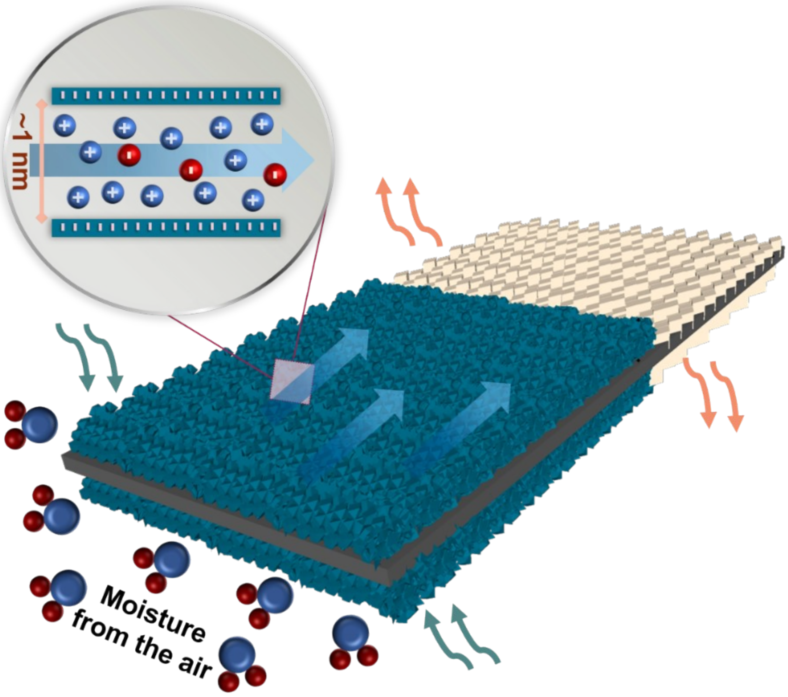

Fig. S19. Illustration of ion migration within the MOFs@FP-CB membrane.

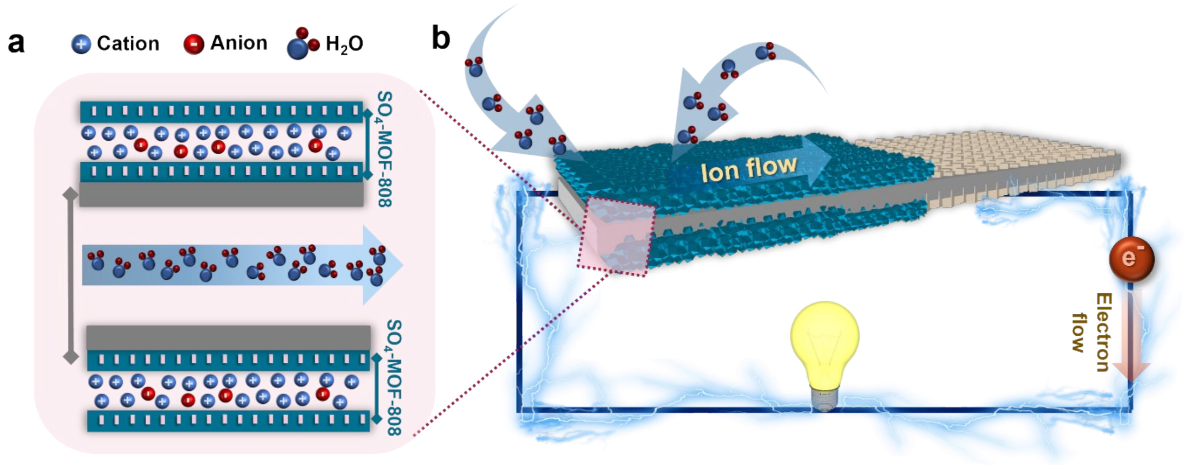

Fig. S20. Schematic diagram of (a) water and ion transport through FP-CB and the nanochannels of SO_4-MOF-808, respectively, and (b) the experimental setup used for electrical measurements.

Table S1. Fabricated MEG membranes along with their material composition.

Membrane	Composition		
	**Carbon black**	**ZIF-8**	**SO_4-MOF-808**
FP	-	-	-
FP-CB	✓	-	-
ZIF-8@FP-CB	✓	✓	-
SO_4-MOF-808@FP-CB	✓	-	✓
MOFs@FP-CB	✓	✓	✓

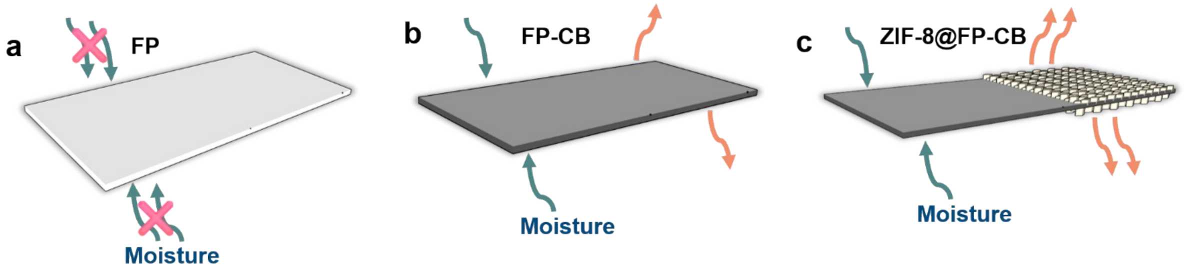

Fig. S21. Schematic illustrations of (a) untreated filter paper, (b) carbon black-treated filter paper, and (c) one-side ZIF-8-coated FP-CB after exposure to a controlled environment, demonstrating limited ability to absorb ambient moisture.

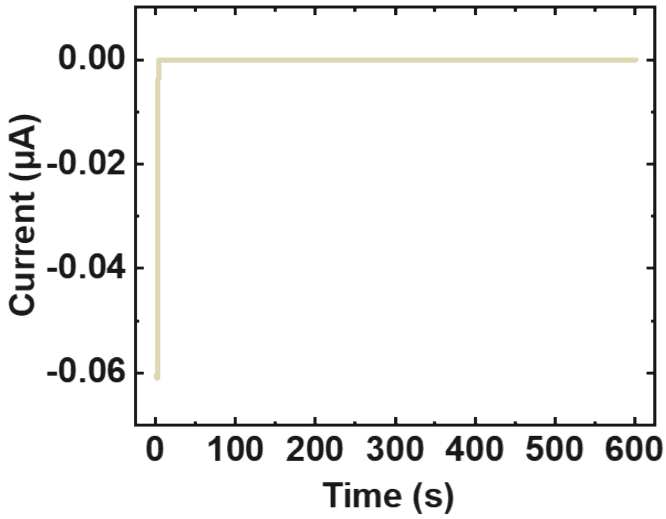

Fig. S22. No measurable current is generated by the pristine filter paper.

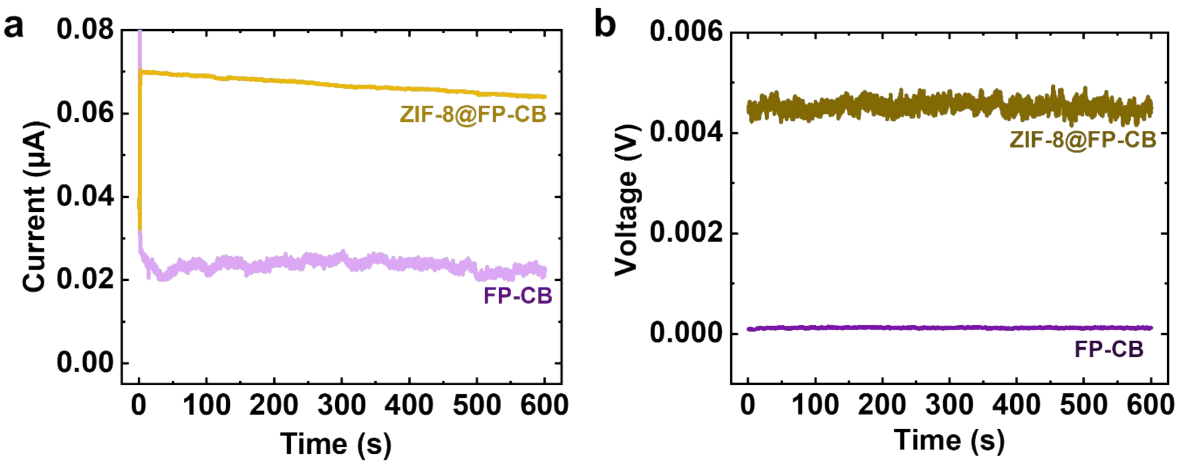

Fig. S23. (a) Current and (b) voltage of the moisture energy generation device using FP-CB and ZIF-8@FP-CB membranes in the absence of an absorbing layer.

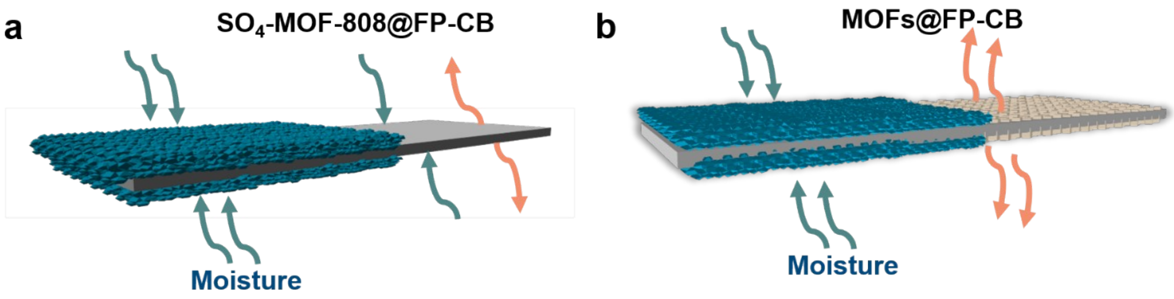

Fig. S24. The presence of hygroscopic SO_4-MOF-808 on one side of the FP-CB membrane facilitates moisture absorption from the air, both (a) without and (b) with the addition of hydrophobic ZIF-8 layer.

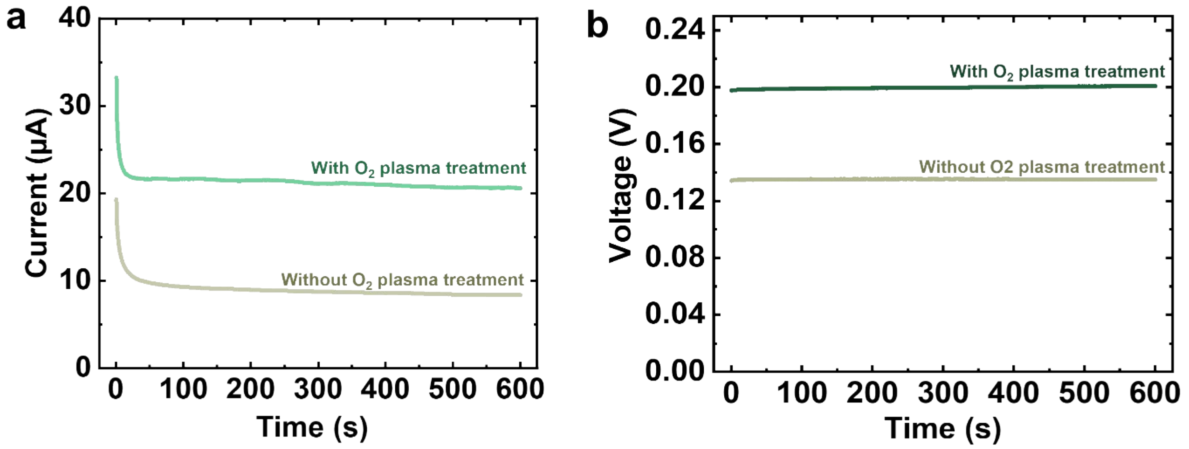

Fig. S25. Influence of O_2 plasma treatment on the extracted (a) current and (b) voltage of the MOFs@FP-CB membrane.

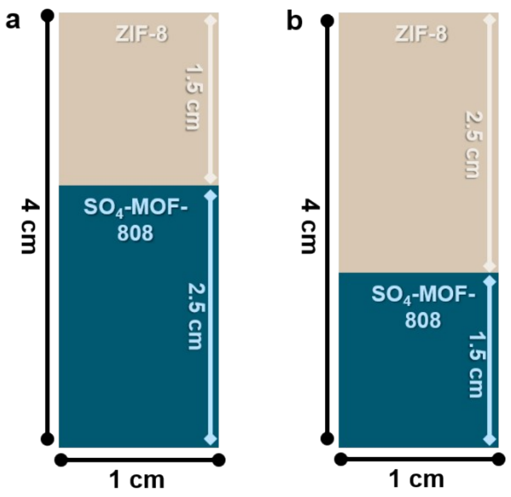

Fig. S26. Two MOFs@FP-CB membrane designs with different MOF ratios. (a) higher absorbing layer of SO_4-MOF-808 and (b) higher evaporating layer of ZIF-8 ratio.

Table S2. As-synthesized MOFs@FP-CB membranes prepared with varying MOF ratios.

Membrane	Length (cm)	
	ZIF-8	SO_4-MOF-808
$MOFs_{2.5}$@FP-CB	1.5	2.5
$MOFs_{1.5}$@FP-CB	2.5	1.5

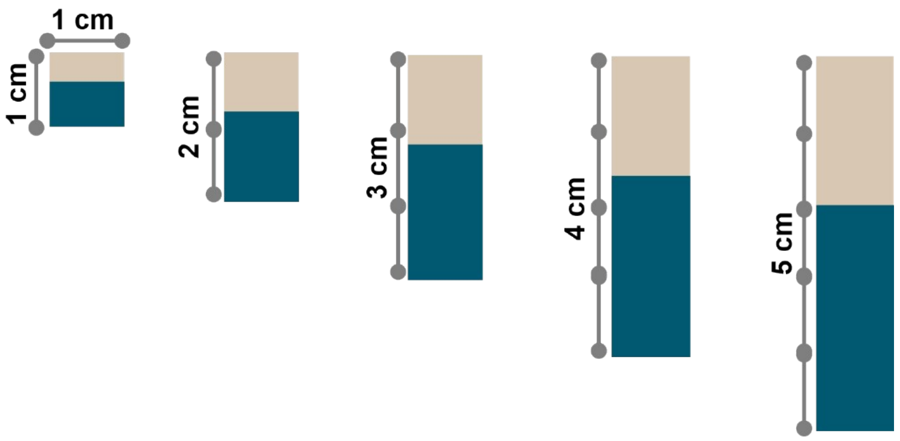

Fig. S27. The length of the MOFs@FP-CB membrane was varied from 1 to 5 cm, while other dimensions were kept constant.

Table S3. The length-dependent characteristic of MOFs@FP-CB membrane.

Membrane	Size (cm)	
	Length	Width
MOFs@FP-CB_1	1	
MOFs@FP-CB_2	2	
MOFs@FP-CB_3	3	1
MOFs@FP-CB_4	4	
MOFs@FP-CB_5	5	

Output power calculation

The captured electrical power can be estimated analogously to a voltaic battery, where approximately one-fourth of the maximum output power (P_{max}),[1] calculated using Equation (S1), is deliverable to an external circuit.

$$P_{\max} = IxV \tag{S1}$$

Accordingly, the generated power density (P) is calculated using Equation (S2):

$$P = \frac{P_{\max}}{4(A)} \tag{S2}$$

where I is the generated current, V is the measured voltage, and A is the membrane area.

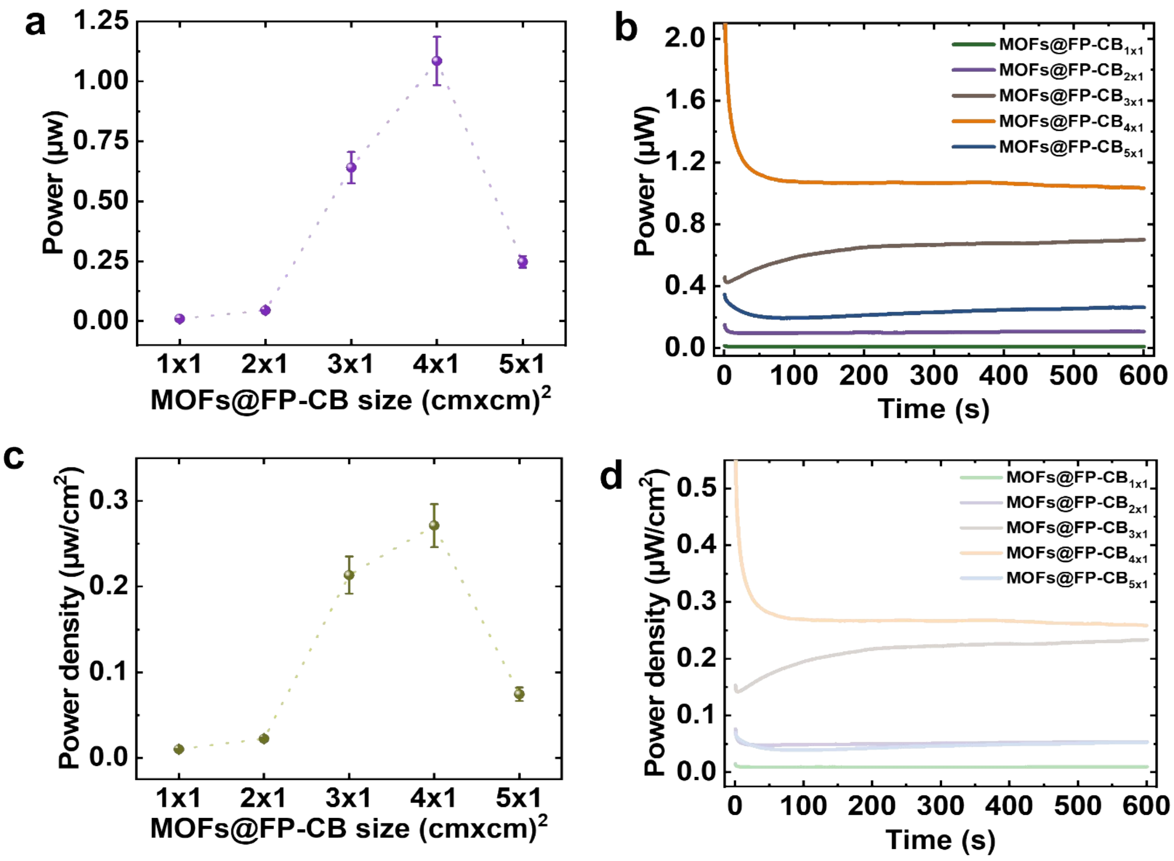

Fig. S28. The calculated (a-b) power and (c-d) power density of the MOFs@FP-CB membrane as function of membrane size.

Table S4. MOFs@FP-CB membranes prepared with varying MOFs coating times.

**Membrane**	**MOFs coating time**	
	**ZIF-8**	**SO_4-MOF-808**
MOFs@FP-CB_1	1	1
MOFs@FP-CB_2	2	2
MOFs@FP-CB_3	3	3
MOFs@FP-CB_4	4	4
MOFs@FP-CB_5	5	5

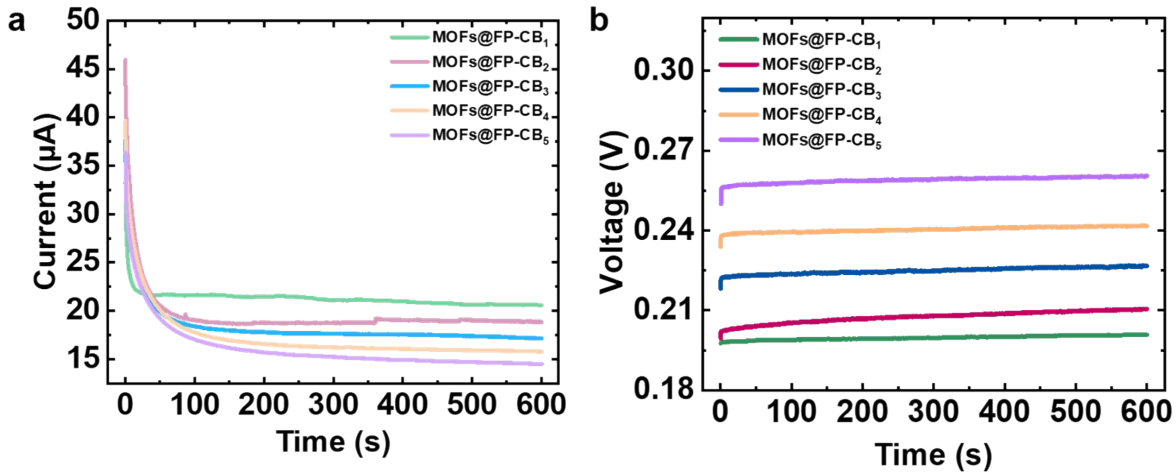

Fig. S29. Effect of MOF layer thickness on device performance. (a) current and (b) voltage.

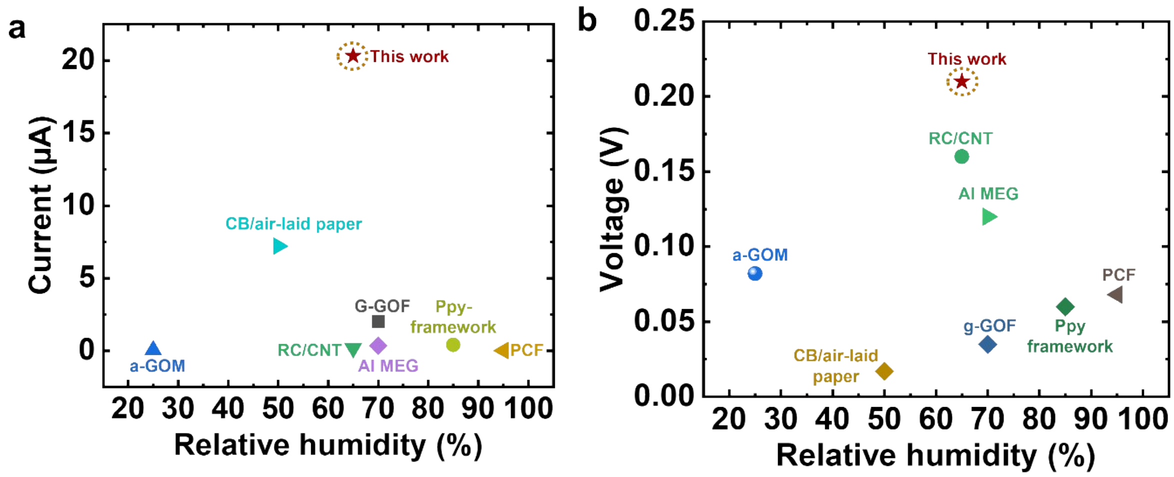

Fig. S30. Comparison of (a) current and (b) voltage generated by the developed MOFs@FP-CB membrane with those of other reported moisture energy generation membrane systems.

Table S5. The comparison of MOFs@FP-CB performance with previous literatures.

Membrane	RH (%)	Temperature (°C)	V_{OC} (V)	I_{SC} (μA)	Reference
g-GOF	70	25	0.035	2	[2]
Ppy framework	85	25	0.060	0.4	[3]
a-GOM	25	25	0.082	0.036	[4]
RC/CNT	65	25	0.16	0.17	[5]
Al MEG	70	25	0.12	0.35	[6]
PCF	95	25	0.068	0.003	[7]
CB/air-laid paper	50	25	0.017	7.2	[8]
MOFs@FP-CB	65	20	0.21	20.3	This work

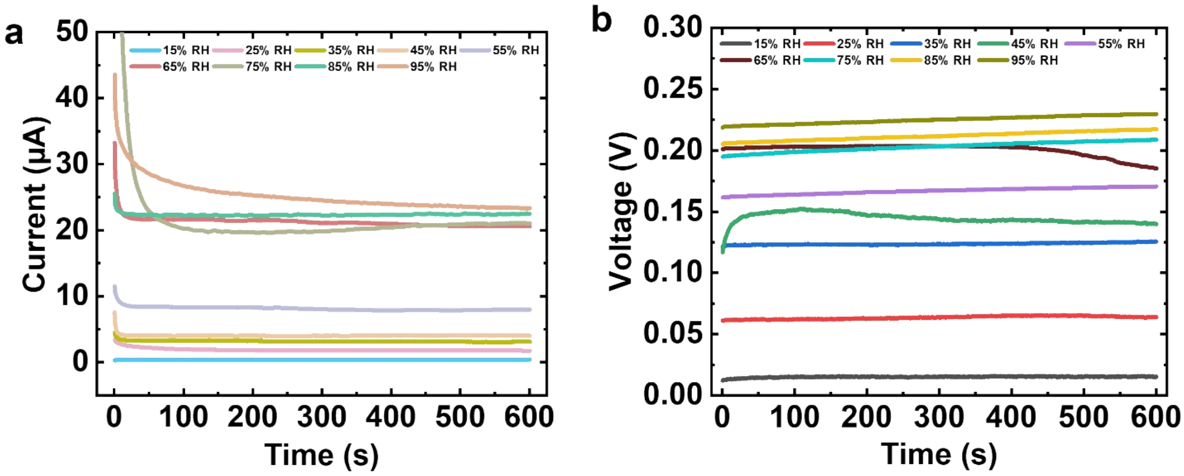

Fig. S31. MOFs@FP-CB can generate (a) current and (b) voltage across a wide range of relative humidity, functioning effectively under both dry and humid conditions.

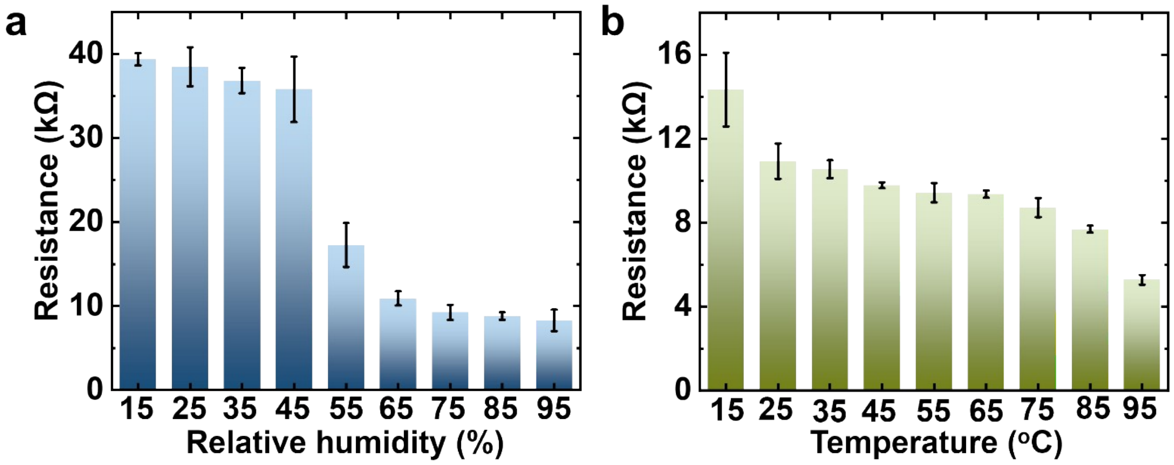

Fig. S32. The calculated resistance of the MOFs@FP-CB membrane as a function of (a) relative humidity and (b) temperature.

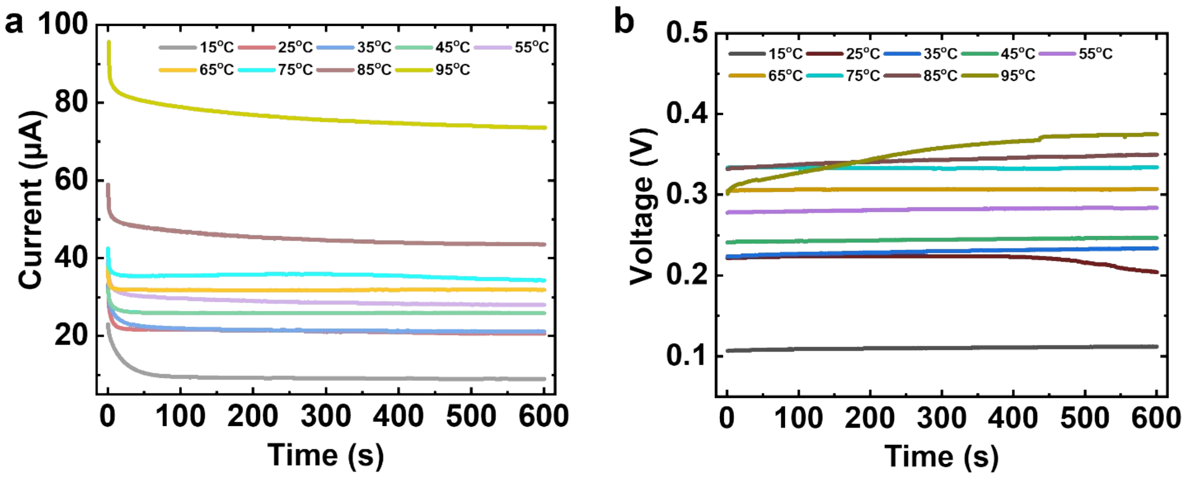

Fig. S33. MOFs@FP-CB can generate (a) current and (b) voltage across a wide temperature range, from cold to hot conditions.

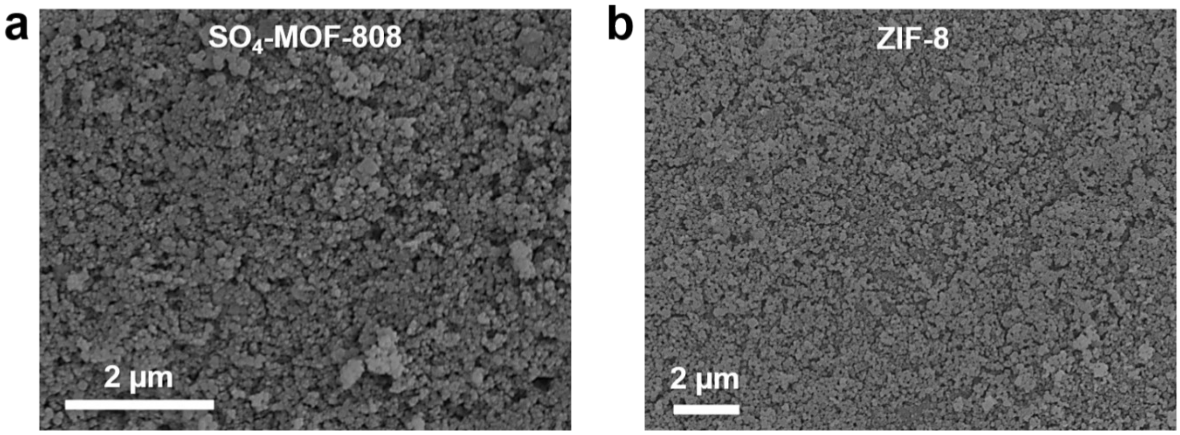

Fig. S34. The FESEM images of (a) SO_4-MOF-808 and (b) ZIF-8 after bending show intact structures and complete coverage of the FP-CB substrate.

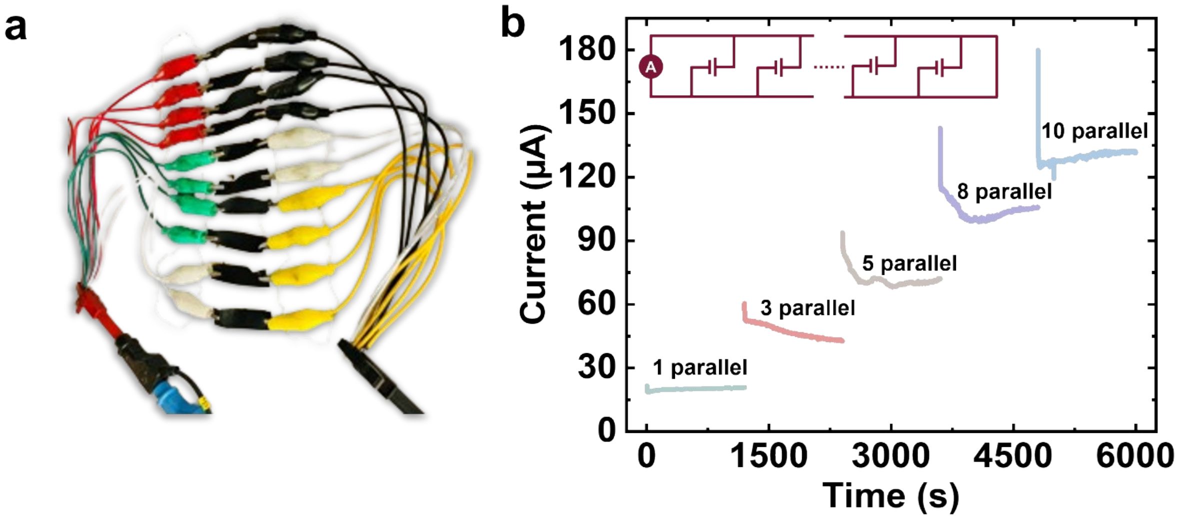

Fig. S35. The current enhancement via parallel connection. (a) Digital photograph of MOFs@FP-CB membranes connected in parallel. (b) Output current as a function of the number of MOFs@FP-CB units connected in series. (Inset: schematic of the circuit configuration).

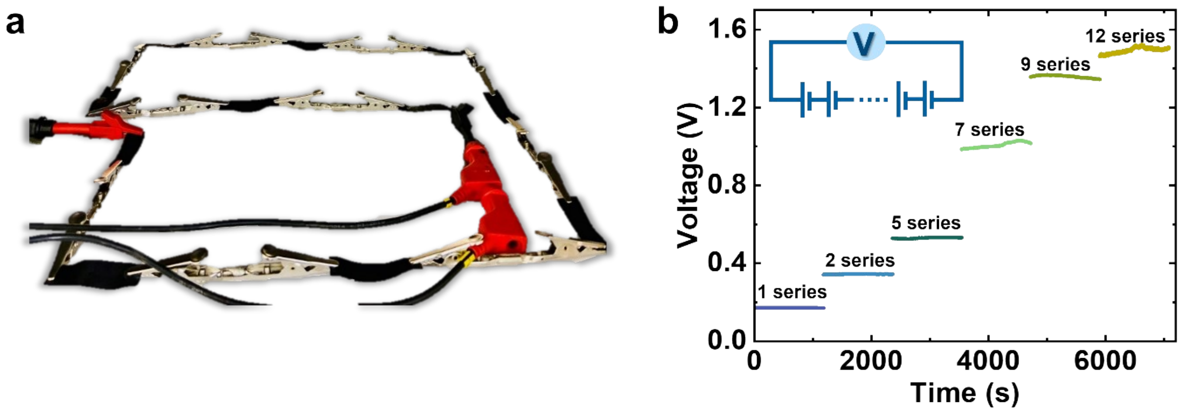

Fig. S36. The voltage scaled up through series connection. (a) Digital photograph of MOFs@FP-CB membranes connected in series. (b) Output voltage as a function of the number of integrated MOFs@FP-CB units connected in series. (Inset: corresponding circuit diagram).

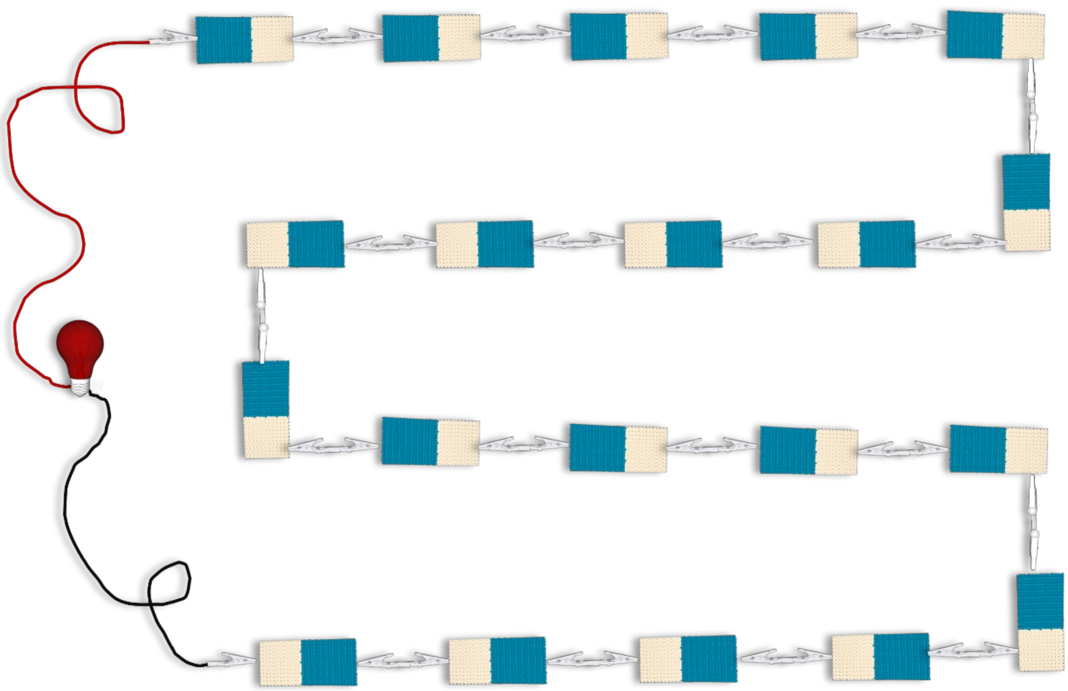

Fig. S37. Schematic representation of a series connection of 20 MOFs@FP-CB devices used to light a red LED.

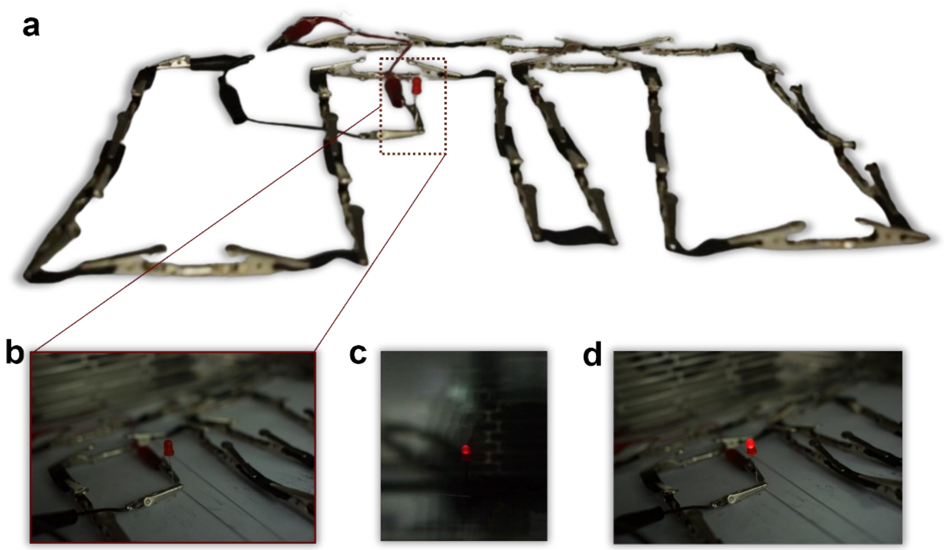

Fig. S38. Demonstration of MOFs@FP-CB devices for powering small electronics. (a) Photograph of 20 MOFs@FP-CB units arranged in series and connected to a red LED and (b) the close-up view of the LED in the off state. (c) LED illuminated by 20 MOFs@FP-CB units connected in series and operating under controlled conditions (25 °C, 65% RH) inside a humidity chamber and (d) LED remains lit for several seconds after exposure to ambient conditions.

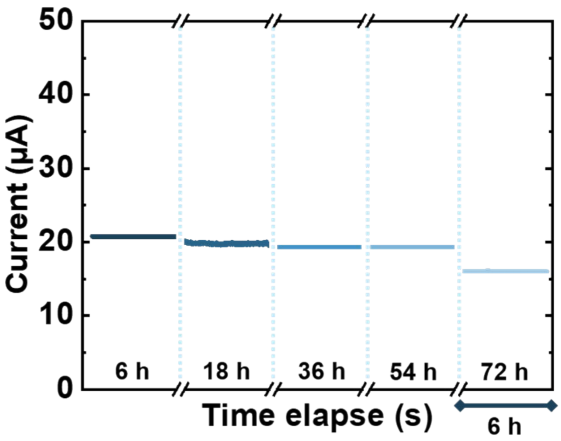

Fig. S39. Continuous current generation stability. The device maintains a stable output current over at least 3 consecutive days of uninterrupted testing.

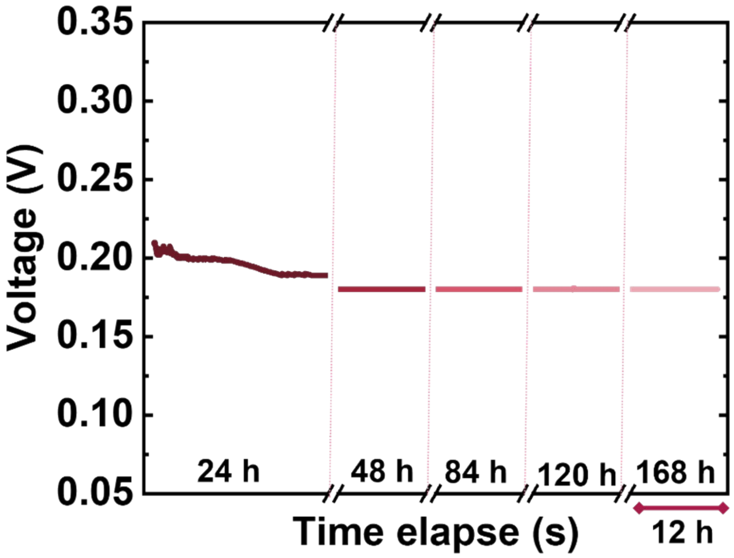

Fig. S40. The long-term voltage stability of the device. The generated voltage remains stable over a continuous 7-day testing period, exhibiting minimal degradation of approximately 10%.

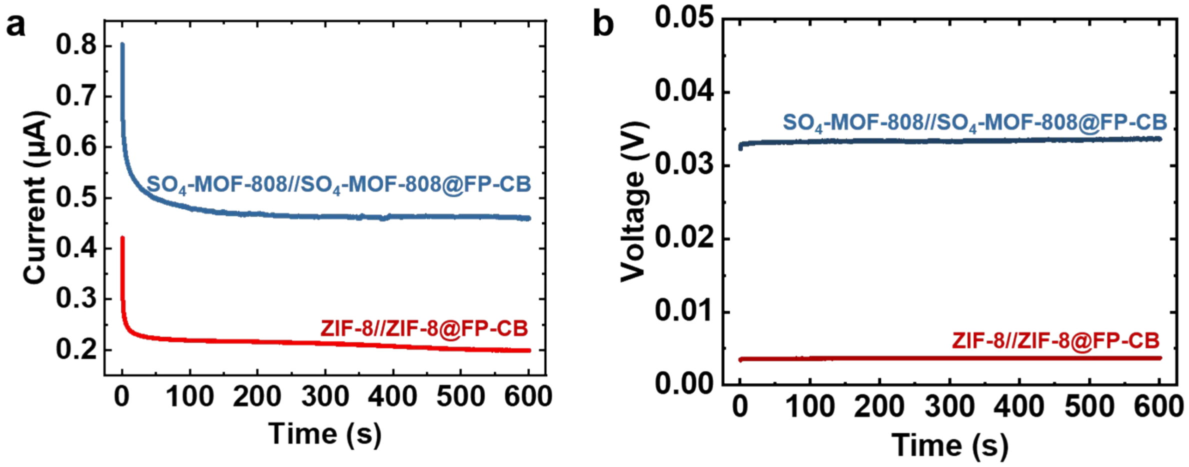

Fig. S41. The symmetric MOF coating of SO_4-MOF-808//SO_4-MOF-808@FP-CB (blue) and ZIF-8//ZIF-8@FP-CB (red) generates low (a) current and (b)voltage.

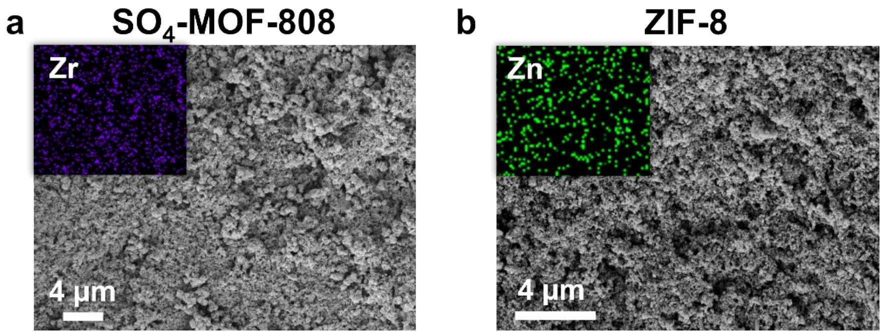

Fig. S42. FESEM images of MOFs@FP-CB after 24 h of continuous operation, showing (a) the SO_4-MOF-808 side and (b) the ZIF-8 side. Insets: Corresponding EDX elemental mappings confirm the sustained presence of Zr and Zn, respectively.

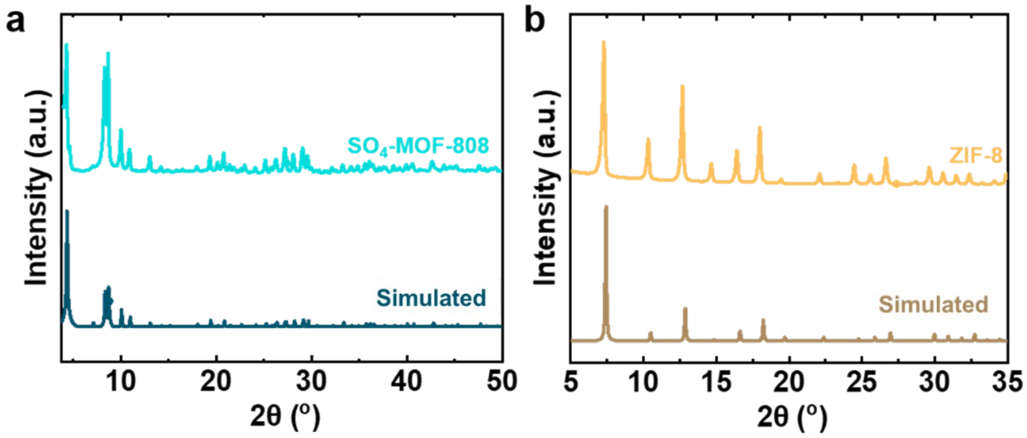

Fig. S43. The post-operation XRD pattern of (a) SO_4-MOF-808 and (b) ZIF-8 showing the same pattern as their simulations based on their reported structures in the crystallographic database.

References

1. C. Guo, H. Tang, P. Wang, Q. Xu, H. Pan, X. Zhao, F. Fan, T. Li and D. Zhao, *Nat. Commun.,* 2024, **15,** 6100.
2. F. Zhao, H. Cheng, Z. Zhang, L. Jiang and L. Qu, *Adv. Mater.,* 2015, **27,** 4351-4357.
3. J. Xue, F. Zhao, C. Hu, Y. Zhao, H. Luo, L. Dai and L. Qu, *Adv. Funct. Mater.,* 2016, **26,** 8784-8792.
4. H. Cheng, Y. Huang, F. Zhao, C. Yang, P. Zhang, L. Jiang, G. Shi and L. Qu, *Energy Environ. Sci.,* 2018, **11,** 2839-2845.
5. J. Chen, Y. Li, Y. Zhang, D. Ye, C. Lei, K. Wu and Q. Fu, *Adv. Funct. Mater.,* 2022, **32,** 2203666.
6. N. Chen, Q. Liu, C. Liu, G. Zhang, J. Jing, C. Shao, Y. Han and L. Qu, *Nano Energy,* 2019, **65,** 104047.
7. K. Liu, P. Yang, S. Li, J. Li, T. Ding, G. Xue, Q. Chen, G. Feng and J. Zhou, *Angew. Chem. Int. Ed.,* 2016, **55,** 8003-8007.
8. Y. Lv, F. Gong, H. Li, Q. Zhou, X. Wu, W. Wang and R. Xiao, *Appl. Energy,* 2020, **279,** 115764.
